# Matching Criterion for Identifiability in Sparse Factor Analysis

**DOI:** 10.1017/psy.2026.10079

**Published:** 2026-01-20

**Authors:** Nils Sturma, Miriam Kranzlmueller, Irem Portakal, Mathias Drton

**Affiliations:** 1 Technical University of Munich, Germany; 2 Ludwig Maximilian University of Munich, Germany; 3 Max Planck Institute for Mathematics in the Sciences, Germany

**Keywords:** covariance matrix, graphical model, latent variables, parameter identification, structural equation model

## Abstract

Factor analysis models explain dependence among observed variables by a smaller number of unobserved factors. A main challenge in confirmatory factor analysis is determining whether the factor loading matrix is identifiable from the observed covariance matrix. The factor loading matrix captures the linear effects of the factors and, if unrestricted, can only be identified up to an orthogonal transformation of the factors. However, in many applications, the factor loadings exhibit an interesting sparsity pattern that may lead to identifiability up to column signs. We study this phenomenon by connecting sparse confirmatory factor analysis models to bipartite graphs and providing sufficient graphical conditions for identifiability of the factor loading matrix up to column signs. In contrast to previous work, our main contribution, the matching criterion, exploits sparsity by operating locally on the graph structure, thereby improving existing conditions. Our criterion is efficiently decidable in time that is polynomial in the size of the graph, when restricting the search steps to sets of bounded size.

## 1 Introduction

In factor analysis, a potentially large set of dependent random variables is modeled as a linear combination of a smaller set of underlying latent (unobserved) factors. Factor analysis is ubiquitously applied in fields, such as econometrics (Aigner et al., 1984; Aßmann et al., 2016; Fan et al., 2008), psychology (Caprara et al., 1993; Ford et al., 1986; Goretzko et al., 2023; Horn, 1965; Reise et al., 2000), epidemiology (de Oliveira Santos et al., 2019; Martínez et al., 1998), and education (Beavers et al., 2013; Schreiber et al., 2006). It also has applications in causality (Pearl, 2000; Spirtes et al., 2000) as a building block for models with latent variables (Barber et al., 2022; Bollen, 1989).

Let $X={\left(X_{v}\right)}_{v\in V}$ be an observed random vector, and let $Y={\left(Y_{h}\right)}_{h\in H}$ be a latent random vector, indexed by finite sets *V* and H , respectively. Factor analysis models postulate that each observed variable $X_{v}$ is a linear function of the *factors* $Y_{h}$ and noise, that is,

$$\begin{array}{c} X=\Lambda Y+\varepsilon , \end{array}$$

where $\Lambda =(\lambda_{vh})\in R^{\left|V|\times |H\right|}$ is an unknown coefficient matrix known as the *factor loading matrix*. The elements of $\varepsilon ={\left(\varepsilon_{v}\right)}_{v\in V}$ are mutually independent noise variables with mean zero and finite, positive variance. We consider orthogonal factor analysis, which means that we assume that the latent factors ${\left(Y_{h}\right)}_{h\in H}$ are mutually independent. The model further assumes that ε is independent of *Y*. Without loss of generality, we fix the scale of the factors such that $\text{Var}Y_{h}=1$ for each factor. The main object of study, the covariance matrix of the observed random vector *X*, is then given by

(1) $$\begin{array}{c} \Sigma :=\text{Cov}X=\Lambda \Lambda^{⊤}+\Omega , \end{array}$$

where Ω is a diagonal matrix with entries $\omega_{vv}=\text{Var}\varepsilon_{v}$ .

Our focus is on *confirmatory* factor analysis (Bollen, 1989, Chapter 7), which pertains to a prespecified model that encodes a scientific hypothesis or was learned previously in an exploratory step. Most interest is typically in models in which the factor loading matrix Λ is sparse. In this article, we assume that the sparsity structure of the factor loading matrix and the number of latent factors |H| are fixed and known. Estimation of the factor loadings in confirmatory analyses has been subject to much controversy, due to the difficulties in determining model identifiability (Long, 1983). A factor analysis model is identifiable if the loading matrix Λ can be recovered from the covariance matrix Σ in (1). If Λ is not identifiable, then its estimates are to some degree arbitrary and standard inferential methods invalid (Cox, 2024; Ximénez, 2006).

In *full* factor analysis, where no restrictions on the factor loading matrix are imposed (Drton et al., 2007), the matrix Λ is never identifiable, due to *rotational invariance*. Indeed, for any orthogonal matrix $Q\in R^{\left|H|\times |H\right|}$ , the product $\tilde{\Lambda }=\Lambda Q$ satisfies

$$\begin{array}{c} \tilde{\Lambda }\tilde{\Lambda^{⊤}}+\Omega =\Lambda QQ^{⊤}\Lambda^{⊤}+\Omega =\Lambda \Lambda^{⊤}+\Omega \end{array}$$

and, thus, $\left(\tilde{\Lambda },\Omega \right)$ determines the same covariance matrix as (Λ,Ω) . For this reason, prior work on full factor analysis focuses on identifiability of $\Lambda \Lambda^{⊤}$ or, equivalently, of Ω . Bekker and ten Berge (1997) characterize generic identifiability, which refers to whether Ω can be uniquely recovered for almost all parameter choices, except for a few corner cases at the so-called Ledermann bound. However, for models with sparsity restrictions on Λ , the situation may improve, allowing for the identifiability of the loading matrix Λ itself up to sign changes of the columns. Identifiability up to column sign is the best we may hope for. If we multiply Λ with a diagonal matrix Ψ with entries in {±1} , then the support of ΛΨ is the same as the support of Λ and it still holds that $\Lambda \Psi \Psi^{⊤}\Lambda^{⊤}=\Lambda \Lambda^{⊤}$ .

Example 1.1. In a re-analysis of a well-known five-dimensional example of Harman (1976, p.14), Trendafilov et al. (2017, Table 1, Column 3) apply $\ell_{1}$ -penalization techniques and infer the following sparsity pattern in the factor loading matrix:

$$\begin{array}{c} \Lambda^{⊤}=\left(\begin{array}{ccccccccc} \lambda_{11} & 0 & \lambda_{31} & \lambda_{41} & \lambda_{51}0 & \lambda_{22} & 0 & \lambda_{42} & \lambda_{52} \end{array}\right). \end{array}$$

This implies that the observed covariance matrix is given by

$$\begin{array}{c} \Sigma =(\sigma_{uv})=\left(\begin{array}{ccccccccccccccccccccc} \omega_{11}+\lambda_{11}^{2} & 0 & \lambda_{11}\lambda_{31} & \lambda_{11}\lambda_{41} & \lambda_{11}\lambda_{51}0 & \omega_{22}+\lambda_{22}^{2} & 0 & \lambda_{22}\lambda_{42} & \lambda_{22}\lambda_{52}\lambda_{11}\lambda_{31} & 0 & \omega_{33}+\lambda_{31}^{2} & \lambda_{31}\lambda_{41} & \lambda_{31}\lambda_{51}\lambda_{11}\lambda_{41} & \lambda_{22}\lambda_{42} & \lambda_{31}\lambda_{41} & \omega_{44}+\lambda_{41}^{2}+\lambda_{52}^{2} & \lambda_{41}\lambda_{51}+\lambda_{42}\lambda_{52}\lambda_{11}\lambda_{51} & \lambda_{22}\lambda_{52} & \lambda_{31}\lambda_{51} & \lambda_{41}\lambda_{51}+\lambda_{42}\lambda_{52} & \omega_{55}+\lambda_{51}^{2}+\lambda_{52}^{2} \end{array}\right). \end{array}$$

For almost every choice of Λ , we have $\sigma_{34}=\lambda_{31}\lambda_{41}\neq 0$ , and the formula

$$\begin{array}{c} \sqrt{\frac{\sigma_{13}\sigma_{14}}{\sigma_{34}}}=\sqrt{\frac{\lambda_{11}\lambda_{31}\lambda_{11}\lambda_{41}}{\lambda_{31}\lambda_{41}}}=\sqrt{\lambda_{11}^{2}}=|\lambda_{11}| \end{array}$$

shows that we can recover the parameter $\lambda_{11}$ up to sign. Given $\left|\lambda_{11}\right|$ , the remaining nonzero parameters of the first column of Λ are easily found, up to $\text{sign}(\lambda_{11})$ . For example,

$$\begin{array}{c} \text{sign}(\lambda_{11})\lambda_{31}=\frac{\lambda_{11}\lambda_{31}}{\left|\lambda_{11}\right|}=\frac{\sigma_{13}}{\left|\lambda_{11}\right|}, \end{array}$$

which is again well-defined for almost all parameter choices. Given $\Lambda_{⋆,1}$ up to sign, it is then possible to identify the second column $\Lambda_{⋆,2}$ up to $\text{sign}(\lambda_{22})$ using similar formulas.

Remark 1.2. If the latent factors are allowed to have arbitrary positive variance instead of fixing $\text{Var}Y_{h}=1$ , then we can only hope for identifiability up to column sign and column scaling of Λ . In this case, the absolute values of the recovered factor loadings within each column can be interpreted as the relative strength of effects.

The fact that sparsity improves identifiability was noted early in the literature, and there exist many methods in exploratory factor analysis that select a model that is as sparse as possible. Kaiser (1958) and Carroll (1953) proposed methods that are still used in modern statistical software (Pedregosa et al., 2011), optimizing over all rotations such that many factor loadings are close to zero and the remaining loadings have a large absolute value. Developing methods for recovering a sparse factor loading matrix remains a very active field of research. Examples include regularization techniques (Goretzko, 2023; Hirose & Konishi, 2012; Lan et al., 2014; Lee & Seregina, 2023; Ning & Georgiou, 2011; Scharf & Nestler, 2019; Trendafilov et al., 2017), rotation methods (Liu et al., 2023), correlation thresholding (Kim & Zhou, 2023), and Bayesian approaches (Conti et al., 2014; Frühwirth-Schnatter et al., 2025; Ročková & George, 2016; Zhao et al., 2016).

In this article, we study identifiability of the factor loading matrix Λ from the population covariance matrix $\Sigma =\Lambda \Lambda^{⊤}+\Omega$ , where the sparsity structure of Λ is fixed and known. Reflecting the problem’s inherent difficulty, the most prominent sufficient condition for identifiability in confirmatory factor analysis is still the criterion of Anderson and Rubin (1956), which certifies identifiability of $\Lambda \Lambda^{⊤}$ . Subsequently, criteria were developed for identifying Λ from $\Lambda \Lambda^{⊤}$ up to column sign (see Williams, 2020 or Bai & Li, 2012, Section 4). Examples include the three-indicator rule of Bollen (1989) and the side-by-side rule of Reilly and O’Brien (1996). However, gaps remain in the existing results. As noted by Hosszejni and Frühwirth-Schnatter (2026), the model given by the sparse matrix

(2) $$\begin{array}{c} \Lambda^{⊤}=\left(\begin{array}{cccccccccccccccc} \lambda_{11} & \lambda_{21} & 0 & \lambda_{41} & 0 & 00 & \lambda_{22} & \lambda_{32} & 0 & \lambda_{52} & 00 & 0 & \lambda_{33} & \lambda_{43} & 0 & \lambda_{63} \end{array}\right) \end{array}$$

is identifiable up to column sign in an almost sure sense, but the criterion of Anderson and Rubin (1956) and the subsequent developments are not able to certify it.

**Figure 1:**
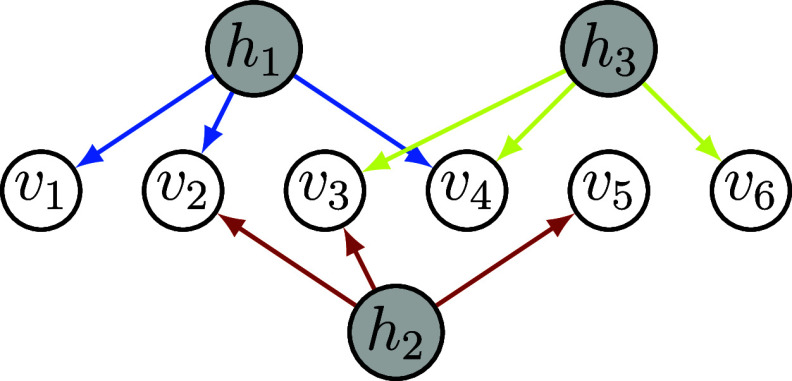
Directed graph encoding the sparsity structure in a factor analysis model. Figure 1 long description.

In contrast to prior work, we take a graphical perspective to specify the sparsity structure in Λ (Lauritzen, 1996; Maathuis et al., 2019). For example, the graph in Figure 1 encodes the sparsity structure in the factor loading matrix given in Equation (2). When an edge h→v is missing in the graph, the corresponding entry $\lambda_{vh}$ is required to be zero. Building on Anderson and Rubin (1956) and Bekker and ten Berge (1997), our new *matching criterion* (and an *extension* thereof) is a purely graphical criterion that exploits sparsity by operating *locally* on the structure of the graph.

Deciding identifiability corresponds to solving the equation system from (1). Since the equations are polynomial in the factor loadings $\lambda_{vh}$ , identifiability is, in principle, always decidable via Gröbner basis methods from computational algebraic geometry (Barber et al., 2022; Garcia-Puente et al., 2010). But the scope of such methods is limited to small graphs as their complexity can grow double exponentially with the size of the graph (Mayr, 1997). In contrast, our new graphical criteria can be checked in polynomial time, provided we restrict a search step to subsets of bounded size.

The organization of the article is as follows. Section 2 formally introduces the concept of generic sign-identifiability, and we revisit the criteria of Anderson and Rubin (1956) and Bekker and ten Berge (1997) in Section 3. Section 4 presents our main results, the matching criterion and its extension. In Section 5, we show that both criteria are decidable in polynomial time. In Section 6, we conduct experiments that demonstrate the performance of our criteria and exemplify how our identifiability criteria are also useful in exploratory factor analysis. The Supplementary Material contains additional results for full factor models (Appendix A of the Supplementary Material), efficient algorithms (Appendix B of the Supplementary Material), all technical proofs (Appendix C of the Supplementary Material), and an explanation of how to decide identifiability using algebraic tools (Appendix D of the Supplementary Material).

## 2 Graphical representation and identifiability

Let G=(V∪H,D) be a directed graph, where *V* and H are finite disjoint sets of observed and latent nodes. We assume that the graph G=(V∪H,D) is bipartite, that is, it only contains edges from latent to observed variables such that D⊆H×V . We refer to such graphs as *factor analysis graphs*. If *G* contains an edge (h,v)∈D , then we also denote this by h→v∈D . The set ch(h)={v∈V:h→v∈D} contains the children of a latent node h∈H , and the set pa(v)={h∈H:h→v∈D} contains the parents of an observed node v∈V .

Each bipartite graph defines a factor analysis model, which for our purposes may be identified with a set of covariance matrices.

Definition 2.1. Let G=(V∪H,D) be a factor analysis graph with |V|=p and |H|=m , and let $R^{D}$ be the space of real p×m matrices $\Lambda =(\lambda_{vh})$ with support *D*, that is, $\lambda_{vh}=0$ if h→v/∈D . The factor analysis model determined by *G* is the image $F(G)=\text{Im}(\tau_{G})$ of the parameterization

$$\begin{array}{c} \backslash split\tau_{G}:R_{>0}^{p}\times R^{D}⟶\backslash operatornamePD(p)(\Omega ,\Lambda )⟼\Omega +\Lambda \Lambda^{⊤}, \end{array}$$

where \operatornamePD(p) is the cone of positive-definite p×p matrices, and $R_{>0}^{p}\subset \backslash operatornamePD(p)$ is the subset of diagonal positive-definite matrices.

Identifiability holds if we can recover Ω and Λ from a given matrix Σ∈F(G) up to column signs of the matrix Λ . To make this precise, we write

$$\begin{array}{c} F_{G}(\Omega ,\Lambda )=\{(\tilde{\Omega },\tilde{\Lambda })\in \Theta_{G}:\tau_{G}(\tilde{\Omega },\tilde{\Lambda })=\tau_{G}(\Omega ,\Lambda )\} \end{array}$$

for the *fiber* of a pair (Ω,Λ) in the domain $\Theta_{G}=R_{>0}^{\left|V\right|}\times R^{D}$ of the parameterization $\tau_{G}$ .

Definition 2.2. A factor analysis graph G=(V∪H,D) is said to be *generically sign-identifiable* if

$$\begin{array}{c} F_{G}(\Omega ,\Lambda )=\{(\tilde{\Omega },\tilde{\Lambda })\in \Theta_{G}:\tilde{\Omega }=\Omega \text{and}\tilde{\Lambda }=\Lambda \Psi \text{for}\Psi \in \{\pm 1{\}}^{\left|H|\times |H\right|}\text{diagonal}\} \end{array}$$

for almost all $(\Omega ,\Lambda )\in \Theta_{G}$ . Moreover, we say that a node h∈H in a factor analysis graph G=(V∪H,D) is *generically sign-identifiable* if it holds for almost all $(\Omega ,\Lambda )\in \Theta_{G}$ that each parameter pair $\left(\tilde{\Omega },\tilde{\Lambda })\in F_{G}(\Omega ,\Lambda \right)$ satisfies $\tilde{\Lambda_{\text{ch}(h),h}}=\Lambda_{\text{ch}(h),h}$ .

In Definition 2.2, “almost all” is meant with respect to the induced Lebesgue measure on $\Theta_{G}$ , considered as an open subset of $R^{\left|V|+|D\right|}$ . If a graph is generically sign-identifiable, then for a factor loading matrix Λ and a diagonal covariance matrix Ω drawn randomly from an absolutely continuous distribution, the resulting covariance matrix of the observable vector *X* will almost surely allow recovery of Λ up to column sign.

Example 2.3. Consider the identification formula for $\left|\lambda_{11}\right|$ in Example 1.1 given by

$$\begin{array}{c} \sqrt{\frac{\sigma_{13}\sigma_{14}}{\sigma_{34}}}=\sqrt{\frac{\lambda_{11}\lambda_{31}\lambda_{11}\lambda_{41}}{\lambda_{31}\lambda_{41}}}. \end{array}$$

This formula does not hold if at least one of the parameters $\lambda_{31}$ and $\lambda_{41}$ is equal to zero. Hence, for such exceptional parameter pairs (Ω,Λ), we cannot establish the correct form of the fiber and identification fails. However, since the set of exceptional pairs forms a Lebesgue measure zero subset of the parameter space, we obtain generic sign-identifiability.

Note that any node *h* with ch(h)=\emptyset is trivially generically sign-identifiable. For later reference, we formally record how generic sign-identifiability of the graph results from generic sign-identifiability of all nodes.

Lemma 2.4. A factor analysis graph G=(V∪H,D) is generically sign-identifiable if and only if all nodes h∈H are generically sign-identifiable.

Remark 2.5. A model can only be generically sign-identifiable if its dimension matches the parameter count |V|+|D| . Recently, Drton et al. (2025) proved upper and lower bounds for the dimension of sparse factor analysis models. The bounds reveal that such models may have dimension strictly smaller than |V|+|D| and, thus, may be non-identifiable. The bounds also show that a necessary condition for a factor analysis graph to be generically sign-identifiable is that each latent node has at least three children.

## 3 Existing criteria

Due to rotational indeterminacy, previous work on identifiability of full factor analysis models focused on identifying the diagonal matrix Ω . If we require that the upper triangle of the matrix Λ is zero, then existing criteria may also yield generic sign-identifiability.

Definition 3.1. A factor analysis graph G=(V∪H,D) satisfies the *zero upper triangular assumption (ZUTA)* if there exists an ordering ≺ on the latent nodes H such that ch(h) is not contained in ${\backslash leftcup}_{\ell ≻h}\text{ch}(\ell )$ for all h∈H . In this case, we say that ≺ is a *ZUTA-ordering* with respect to *G*.

ZUTA ensures that the rows and columns of the factor loading matrix Λ can be permuted such that the upper triangle of the matrix is zero. Note that ZUTA eliminates rotational indeterminacy. That is, if it holds that $\Sigma -\Omega =\tilde{\Lambda }\tilde{\Lambda^{⊤}}$ for a matrix $\tilde{\Lambda }$ that is zero upper triangular, i.e., $\tilde{\Lambda_{ij}}=0$ for i<j , then it follows from the uniqueness of the Cholesky decomposition that $\tilde{\Lambda }$ is unique up to column sign, i.e., $\tilde{\Lambda }=\Lambda \Psi$ for a fixed matrix Λ and a diagonal matrix Ψ with entries in {±1} .

If a factor analysis graph satisfies the ZUTA condition, then there is an observed node $v_{h}\in \text{ch}(h)$ for each h∈H such that $v_{h}\in \text{ch}(h)$ and $v_{h}/\in {\backslash leftcup}_{\ell ≻h}\text{ch}(\ell )$ . In particular, it is a necessary condition for ZUTA that there is at least one observed node that only has one latent parent.

**Figure 2:**
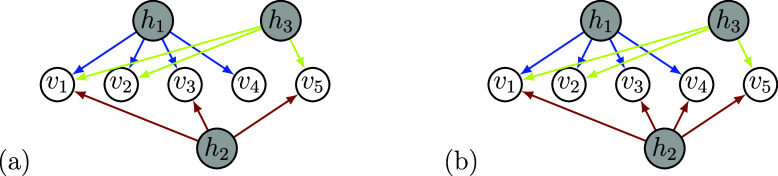
Two factor analysis graphs. Graph (a) satisfies ZUTA while graph (b) does not. Figure 2 long description.

Example 3.2. The graph in Figure 2a satisfies ZUTA with the ordering $h_{1}≺h_{2}≺h_{3}$ , since $v_{4}\in \text{ch}(h_{1})$ but $v_{4}/\in \text{ch}(h_{2})\cup \text{ch}(h_{3})$ , and $v_{3}\in \text{ch}(h_{2})$ but $v_{3}/\in \text{ch}(h_{3})$ . However, the graph in Figure 2b does not satisfy ZUTA as no observed node has only one parent.

Remark 3.3. ZUTA is equivalent to the *generalized lower triangular assumption* introduced in Frühwirth-Schnatter et al. (2025), which operates directly on the matrix Λ . ZUTA refers to the graph, which is useful to present our graphical criteria in Section 4.

If we consider graphs that satisfy ZUTA, many criteria in the literature directly yield generic sign-identifiability. The most prominent condition for identifiability is still the criterion of Anderson and Rubin (1956). Since it is originally stated as a pointwise condition, it is also applicable to sparse graphs. To state the result one obtains, we treat the entries of Λ as indeterminates and say that a submatrix is generically of rank *k* if it has rank *k* for almost all choices of $\Lambda \in R^{D}$ . Under the assumption that a graph satisfies ZUTA, Theorem 5.1 in Anderson and Rubin (1956) then translates to the following sufficient condition for generic sign-identifiability.

Theorem 3.4 **(AR-identifiability)**

Let G=(V∪H,D) be a factor analysis graph that satisfies ZUTA. Then, *G* is generically sign-identifiable if for any deleted row of $\Lambda =(\lambda_{vh})\in R^{D}$ , there remain two disjoint submatrices that are generically of rank |H| .

If generic sign-identifiability can be proven by applying Theorem 3.4 for a factor analysis graph, then we say that the graph is *AR-identifiable*.

**Figure 3:**
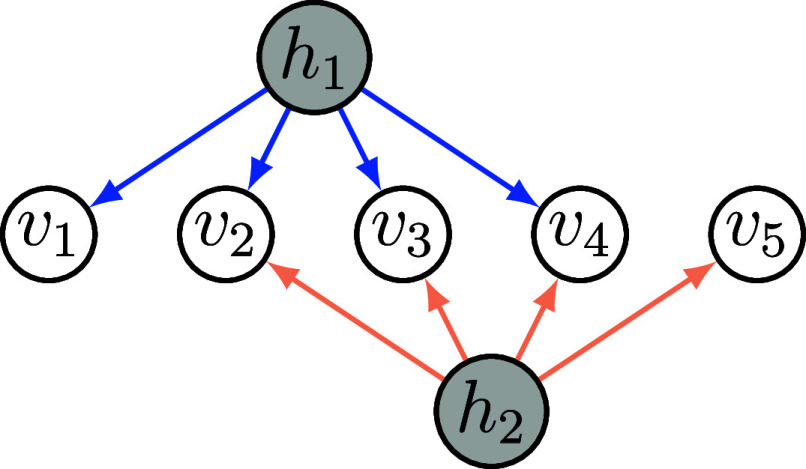
AR-identifiable factor analysis graph.

Example 3.5. The graph in Figure 3 gives rise to the transpose of $\Lambda \in R^{D}$ given by

$$\begin{array}{c} \Lambda^{⊤}=\left(\begin{array}{cc} \lambda_{v_{1}h_{1}}\\ \lambda_{v_{2}h_{1}}\\ \lambda_{v_{3}h_{1}}\\ \lambda_{v_{4}h_{1}}\\ 0 & 0\\ \lambda_{v_{3}h_{2}}\\ \lambda_{v_{3}h_{2}}\\ \lambda_{v_{4}h_{2}}\\ \lambda_{v_{5}h_{2}} \end{array}\right). \end{array}$$

Deleting any row of Λ leaves four rows that can always be split into two 2×2 matrices that generically have rank 2. Hence, the graph is AR-identifiable.

AR-identifiability requires |V|≥2|H|+1 . For general *full* factor analysis models, Bekker and ten Berge (1997) solve the problem of generic identifiability (up to orthogonal transformation) in all but certain edge cases. However, the generic nature of their condition implies sign-identifiability results only for dense ZUTA graphs, in which only a permuted upper triangle vanishes.

Definition 3.6. A *full-ZUTA graph* is a factor analysis graph G=(V∪H,D) that satisfies ZUTA but contains all other possible edges. That is, there is an ordering ≺ on the latent nodes $H=\{h_{1},\cdots ,h_{m}\}$ such that $h_{1}≺\cdots ≺h_{m}$ , with the property that $\text{ch}(h_{1})=V$ and $\text{ch}(h_{i+1})=\text{ch}(h_{i})⧵\{v_{i}\}$ for some child $v_{i}\in \text{ch}(h_{i})$ .

**Figure 4:**
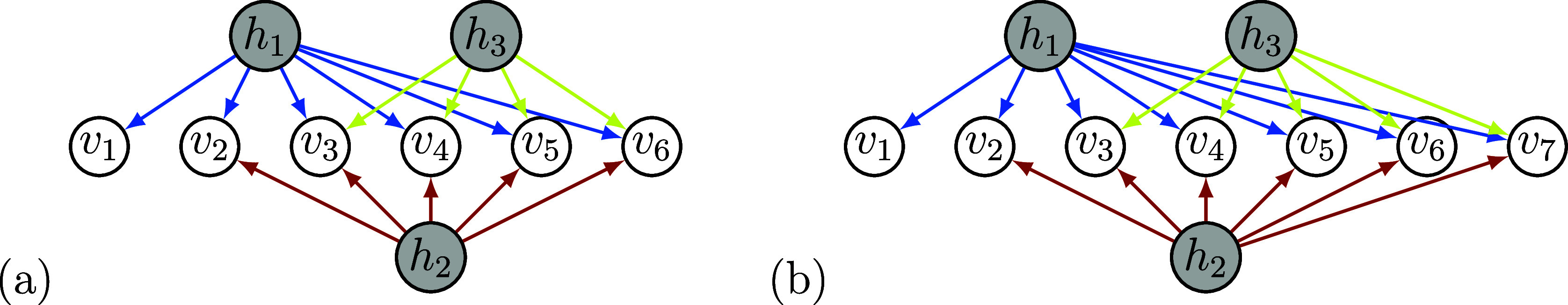
Two full-ZUTA graphs. Figure 4 long description.

As an example, Figure 4a displays the full-ZUTA graph on three latent and six observed nodes. For full-ZUTA graphs, the criterion from Bekker and ten Berge (1997) directly translates into the following sufficient condition for generic sign-identifiability.

Theorem 3.7 **(BB-identifiability)**

Let G=(V∪H,D) be a full-ZUTA graph. Then, *G* is generically sign-identifiable if |V|+|D|<(|V|+12) .

If a full-ZUTA graph is generic sign-identifiability by Theorem 3.7, then we term the graph *BB-identifiable*. Note that |V|+|D|=|V|(|H|+1)−(|H|2) in a full-ZUTA graph. If |V|+|D|>(|V|+12) , then the parameter count is larger than the dimension of the ambient space of symmetric matrices, and full-ZUTA graphs are not generically sign-identifiable (recall Remark 2.5). Hence, the only remaining open cases where identifiability of full-ZUTA graphs is unknown are models “at the Ledermann bound,” where |V|+|D|=(|V|+12) .

Example 3.8. Figure 4 shows two full-ZUTA graphs. Graph (b) is BB-identifiable because |V|+|D|=24<28=(7+12). Graph (a), on the other hand, has |V|+|D|=21=(6+12). As already noted by Wilson and Worcester (1939), the fiber for graph (a), with |V|=6 and |H|=3 , generically contains two diagonal matrices and two corresponding factor loading matrices together with their symmetries given by the sign changes of the columns.

**Figure 5:**
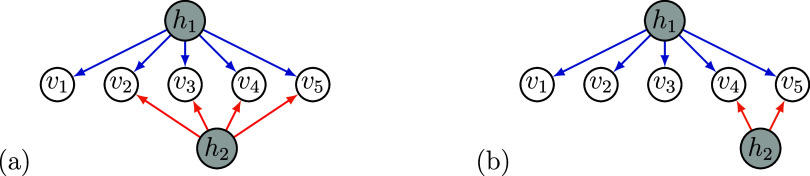
Full-ZUTA graph and a sparse subgraph.

Remark 3.9. Generic sign-identifiability of full-ZUTA graphs does not imply identifiability of sparse subgraphs, since the models corresponding to subgraphs might be non-generic points in the model given by the full-ZUTA graph. For example, consider the full-ZUTA graph in Figure 5a that is generically sign-identifiable by Theorem 3.7. The graph in Figure 5b is a sparse subgraph. Since in this graph $|\text{ch}(h_{2})|<3$ , it follows that the model has not expected dimension and is hence not generically sign-identifiable (recall Remark 2.5).

The following example shows two graphs that are generically sign-identifiable but no known general criterion is able to certify it.

**Figure 6:**
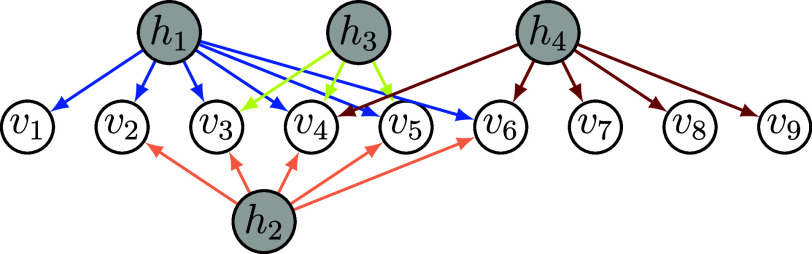
Sparse factor analysis graphs that is not AR-identifiable nor BB-identifiable. Figure 6 long description.

Example 3.10. The loading matrix for the graph in Figure 6 has transpose

$$\begin{array}{c} \Lambda^{⊤}=\left(\begin{array}{ccccccccccccccccccccccccccccccccc} \lambda_{v_{1}h_{1}} & \lambda_{v_{2}h_{1}} & \lambda_{v_{3}h_{1}} & \lambda_{v_{4}h_{1}} & \lambda_{v_{5}h_{1}} & \lambda_{v_{6}h_{1}} & 0 & 0 & 00 & \lambda_{v_{2}h_{2}} & \lambda_{v_{3}h_{2}} & \lambda_{v_{4}h_{2}} & \lambda_{v_{5}h_{2}} & \lambda_{v_{6}h_{2}} & 0 & 0 & 00 & 0 & \lambda_{v_{3}h_{3}} & \lambda_{v_{4}h_{3}} & \lambda_{v_{5}h_{3}} & 0 & 0 & 0 & 00 & 0 & 0 & \lambda_{v_{4}h_{4}} & 0 & \lambda_{v_{6}h_{4}} & \lambda_{v_{7}h_{4}} & \lambda_{v_{8}h_{4}} & \lambda_{v_{9}h_{4}} \end{array}\right). \end{array}$$

The graph is not BB-identifiable as it is not full-ZUTA. To see that it is not AR-identifiable, delete the row of Λ indexed by $v_{4}$ . If we form two 4×4 -matrices out of the remaining eight rows, then one of these matrices has to contain at least two rows indexed by $v_{7}$ , $v_{8},$ or $v_{9}$ . This matrix has at most rank three, which disproves AR-identifiability. Another example that is neither AR- nor BB-identifiable is the graph in Figure 1. Using the criteria we develop in the next section, we can certify identifiability of both graphs (see also Example 4.9).

Finally, we note that BB-identifiability subsumes AR-identifiability for full-ZUTA graphs.

Corollary 3.11. Let G=(V∪H,D) be a full-ZUTA graph with |H|≥2 latent nodes that is AR-identifiable. Then, *G* is also BB-identifiable.

However, there are full-ZUTA graphs that are BB- but not AR-identifiable. The smallest example has |V|=8 observed nodes and |H|=4 latent nodes.

## 4 Main identifiability results

In this section, we derive novel graphical criteria that are sufficient for generic sign-identifiability in sparse factor analysis graphs. As we will show, the criteria strictly generalize AR- and BB-identifiability for ZUTA graphs and are capable of certifying identifiability of models not covered by the AR- nor BB-criterion.

### 4.1 Matching criterion

Our first criterion takes the form of a recursive procedure and is based on a graphical extension of the Anderson–Rubin criterion that can be applied locally at a given node. In the AR criterion, for each observed node v∈V , we need to find disjoint sets U,W⊆V⧵{v} with |U|=|W|=|H| such that $\backslash det(\Lambda_{U,H})\neq 0$ and $\backslash det(\Lambda_{W,H})\neq 0$ . This is equivalent to $\backslash det({\Lambda \Lambda^{⊤}}_{U,W})\neq 0$ . Our main idea is to derive, and locally apply, a modified version of the AR criterion that also considers sets U,W with cardinality smaller than |H| . In doing so, we need to ensure that $\backslash det({\Lambda \Lambda^{⊤}}_{U,W})\neq 0$ , i.e., we need to characterize when minors of $\Lambda \Lambda^{⊤}$ vanish. This can be achieved via the concept of trek separation (Sullivant et al., 2010) and leads to the following definition.

Definition 4.1. Let G=(V∪H,D) be a factor analysis graph, and let A,B⊆V be two subsets of equal cardinality, |A|=|B|=n . A *matching* of *A* and *B* is a system $\Pi =\{\pi_{1},\cdots ,\pi_{n}\}$ consisting of paths of the form

$$\begin{array}{c} \pi_{i}:v_{i}\leftarrow h_{i}\to w_{i},\;i=1,\cdots ,n, \end{array}$$

where all $h_{i}\in H$ , and $\{v_{1},\cdots ,v_{n}\}=A$ and $\{w_{1},\cdots ,w_{n}\}=B$ . A matching is *intersection-free* if the $h_{i}$ are all distinct, and a matching *avoids* L⊆H if $L\cap \{h_{1},\cdots ,h_{n}\}=\backslash emptyset$ .

Example 4.2. Consider the sets $A=\{v_{2},v_{3}\}$ and $B=\{v_{4},v_{5}\}$ in the graph from Figure 5a. The system $\left\{v_{2}\leftarrow h_{1}\to v_{3},v_{4}\leftarrow h_{2}\to v_{5}\right\}$ is an intersection-free matching of *A* and *B*. If instead $A=\{v_{1},v_{2},v_{3}\}$ and $B=\{v_{1},v_{4},v_{5}\}$ , then any matching between *A* and *B* has an intersection. An example is given by the set of paths $\left\{v_{1}\leftarrow h_{1}\to v_{1},v_{2}\leftarrow h_{1}\to v_{3},v_{4}\leftarrow h_{2}\to v_{5}\right\}$ that intersects in the latent node $h_{1}$ .

Our main tool is a lemma that considers determinants of submatrices of $\Lambda \Lambda^{⊤}$ for $\Lambda \in R^{D}$ . Here, we view the determinant as a polynomial in the indeterminates $\lambda_{vh}$ , that is, we view it as an algebraic object without reference to its evaluation at specific values.

Lemma 4.3. Let G=(V∪H,D) be a factor analysis graph, and let $\Lambda \in R^{D}$ . For two subsets A,B⊆V of equal cardinality, $\backslash det({\Lambda \Lambda^{⊤}}_{A,B})$ is not the zero polynomial if and only if there is an intersection-free matching of *A* and *B*.

Applying Lemma 4.3 to Anderson and Rubin’s theorem yields the following corollary.

Corollary 4.4. Let G=(V∪H,D) be a factor analysis graph that satisfies ZUTA. Then, *G* is AR-identifiable if and only if for all v∈V , there exist two disjoint sets W,U⊆V⧵{v} with |W|=|U|=|H| such that there is an intersection-free matching between *W* and *U*.

Example 4.5. We saw in Example 3.5 that the graph in Figure 3 is AR-identifiable. Corollary 4.4 allows us to certify AR-identifiability in a fully graphical way without relating to the factor loading matrix. For example, for node $v_{5}$ , we observe that the two sets $U=\{v_{1},v_{2}\}$ and $W=\{v_{3},v_{4}\}$ have intersection-free matching $\{v_{1}\leftarrow h_{1}\to v_{2},v_{3}\leftarrow h_{2}\to v_{4}\}.$

Remark 4.6. The use of matchings to verify AR-identifiability also appears in recent work of Hosszejni and Frühwirth-Schnatter (2026, Proposition 2) who make a connection between computing classical maximal matchings in bipartite graphs and verifying AR-identifiability. They consider matchings that are defined on *duplicate bipartite* graphs, which are constructed by first duplicating all latent nodes of the original graph and then duplicating the edges connecting these new latent nodes to the original observed nodes. The criterion of Hosszejni and Frühwirth-Schnatter (2026) then establishes AR-identifiability by checking whether the duplicate bipartite graph admits a maximal matching that covers all latent nodes, both the original and their duplicates. However, this approach is not feasible when we modify Corollary 4.4 to be locally applicable, as we do next. The reason is that if not all latent nodes are part of the matching, we do not know a priori which nodes we should consider in the bipartite graph. Therefore, we consider intersection-free matchings defined with respect to the original factor analysis graph.

We are now ready to define our new matching criterion, which operates “node-wise” and considers generic sign-identifiability for individual latent nodes h∈H .

Definition 4.7. Fix a latent node h∈H in the factor analysis graph G=(V∪H,D) . A tuple $(v,W,U,S)\in V\times 2^{V}\times 2^{V}\times 2^{H⧵\{h\}}$ satisfies the *matching criterion* with respect to *h* if

-
pa(v)⧵S={h} and v/∈W∪U ;
- *W* and *U* are disjoint, nonempty sets of equal cardinality;
- there exists an intersection-free matching of *W* and *U* that avoids *S*;
- there is no intersection-free matching of {v}∪W and {v}∪U that avoids *S*.

If (v,W,U,S) satisfies the matching criterion with respect to *h*, then Condition (iii) ensures $\backslash det({\Lambda \Lambda^{⊤}}_{W,U})\neq 0$ , and Condition (iv) ensures $\backslash det({\Lambda \Lambda^{⊤}}_{\{v\}\cup W,\{v\}\cup U})=0$ after removing the nodes in *S* from the graph. The Laplace expansion of determinants then allows us to find a rational formula for $\lambda_{vh}^{2}$ in terms of the entries of the covariance matrix. We can thus identify $\lambda_{vh}$ up to sign. Having identified parameter $\lambda_{vh}$ for one child v∈ch(h) , it is easy to certify sign-identifiability of *h*, i.e., to identify the remaining parameters $\lambda_{uh}$ for u∈ch(h)⧵{v} up to the same sign. This is formalized in our first main result.

Theorem 4.8 **(M-identifiability)**

Let G=(V∪H,D) be a factor analysis graph, and fix a latent node h∈H . Suppose that the tuple $(v,W,U,S)\in V\times 2^{V}\times 2^{V}\times 2^{H⧵\{h\}}$ satisfies the matching criterion with respect to *h*. If all nodes ℓ∈S are generically sign-identifiable, then *h* is generically sign-identifiable.

Theorem 4.8 provides a way to recursively certify generic sign-identifiability of a factor analysis graph by checking whether all nodes h∈H are generically sign-identifiable (recall Lemma 2.4). If generic sign-identifiability can be certified recursively by Theorem 4.8, then we call the factor analysis graph *M-identifiable*. The details of an efficient algorithm to check M-identifiability using max-flow techniques are described in Appendix B of the Supplementary Material.

**Figure 7:**
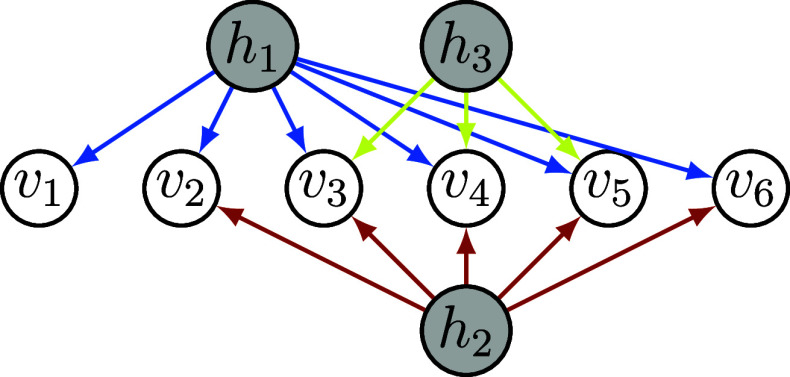
M-identifiable sparse factor analysis graph.

Example 4.9. The factor analysis graph in Figure 7 is not AR-identifiable since |V|=2|H| . However, it is M-identifiable. We recursively check all latent nodes $H=\{h_{1},h_{2},h_{3}\}$ .

1. Take $v=v_{1}$ , S=\emptyset , $U=\{v_{2},v_{6}\}$ , and $W=\{v_{3},v_{4}\}$ . Conditions (i) and (ii) are easily checked, and for (iii), an intersection-free matching is given by $\left\{v_{2}\leftarrow h_{1}\to v_{3},v_{6}\leftarrow h_{2}\to v_{4}\right\}$ . To verify (iv), note that $\text{pa}(\{v\}\cup U)\cap \text{pa}(\{v\}\cup W)=\{h_{1},h_{2}\}$ , which implies that there cannot exist an intersection-free matching of {v}∪U and {v}∪W .
2. Take $v=v_{2}$ , $S=\{h_{1}\}$ , $U=\{v_{3}\}$ , and $W=\{v_{6}\}$ . The matching $\left\{v_{3}\leftarrow h_{2}\to v_{6}\right\}$ is intersection-free, and $\left(\text{pa}(\{v\}\cup U)\cap \text{pa}(\{v\}\cup W))⧵S=\{h_{2}\right\}$ implies that (iv) holds.
3. Take $v=v_{3}$ , $S=\{h_{1},h_{2}\}$ , $U=\{v_{4}\}$ , and $W=\{v_{5}\}$ . The matching $\left\{v_{4}\leftarrow h_{3}\to v_{5}\right\}$ is intersection-free, and $\left(\text{pa}(\{v\}\cup U)\cap \text{pa}(\{v\}\cup W))⧵S=\{h_{3}\right\}$ implies that (iv) holds.

Note that the graphs in Figures 1 and 6 are also M-identifiable, which can be seen similarly.

Next, we show that M-identifiability subsumes AR-identifiability.

Corollary 4.10. Let G=(V∪H,D) be a factor analysis graph that satisfies ZUTA. Then:

- If *G* is AR-identifiable, then it is also M-identifiable.
- If *G* is full-ZUTA, then *G* is AR-identifiable if and only if it is M-identifiable.

Even though M-identifiability subsumes AR-identifiability, it can also only establish identifiability of graphs that satisfy ZUTA.

Corollary 4.11. Let G=(V∪H,D) be a factor analysis graph that is M-identifiable. Then, the factor analysis graph *G* satisfies ZUTA.

### 4.2 Extension of the matching criterion

By Corollary 4.10, M-identifiability subsumes AR-identifiability. However, it does not subsume BB-identifiability. For example, the full-ZUTA graph on |V|=8 observed nodes and |H|=4 latent nodes is BB- but not M-identifiable. We now provide a second criterion that can certify generic sign-identifiability of a set of latent nodes in a way that generalizes BB-identifiability. It operates by searching locally for full-ZUTA subgraphs $\tilde{G}=(\tilde{V},\tilde{D})$ that satisfy the condition |V~|+|D~|<(|V~|+12) . Combining both criteria then yields an extension of the matching criterion. We start by defining the necessary concepts.

Definition 4.12. For a set B⊆V of observed nodes, the set of *joint parents* of pairs in *B* is given by

$$\begin{array}{c} \text{jpa}(B)=\{h\in \text{pa}(u)\cap \text{pa}(v):u,v\in B,u\neq v\}. \end{array}$$

Moreover, for another set S⊆V , we say that an ordering ≺ on the set *S* is a *B-first-ordering* if, for two elements v,w∈S , it holds that v≺w whenever v∈B∩S and w∈S⧵B .

Said differently, a *B*-first-ordering on a set of nodes *S* is a block-ordering such that all elements in *B* come first.

Example 4.13. Consider the graph in Figure 7, and let $B=\{v_{1},v_{2},v_{3}\}$ . The joint parents are given by $\text{jpa}(B)=\{h_{1},h_{2}\}$ . Moreover, for $S=\{v_{1},v_{2},v_{4},v_{5}\}$ , an example of a *B*-first ordering is given by $v_{2}≺v_{1}≺v_{5}≺v_{4}$ .

We now define a criterion that generalizes BB-identifiability. For A⊆V∪H , we write $GA=(A,D_{A})$ for the *induced* subgraph of G=(V∪H,D) . The edge set $D_{A}=\{h\to v\in D:h,v\in A\}$ includes precisely those edges in *D* that have both endpoints in *A*.

Definition 4.14. Let G=(V∪H,D) be a factor analysis graph. We say that the tuple $(B,S)\in 2^{V}\times 2^{H}$ satisfies the *local BB-criterion* if

- the induced subgraph $\tilde{G}=GB\cup (\text{jpa}(B)⧵S)$ is a full-ZUTA graph;
- for all h∈jpa(B)⧵S , there is a *B*-first-ordering ${≺}_{h}$ on ch(h) such that for all v∈ch(h)⧵B, there is u∈ch(h) with $u{≺}_{h}v$ and $\text{jpa}(\{v,u\})⧵S\subseteq \{\ell \in \text{jpa}(B)⧵S:\ell {⪯}_{\text{ZUTA}}h\}$ , where ${≺}_{\text{ZUTA}}$ is the unique ZUTA-ordering on jpa(B)⧵S induced by $\tilde{G}$ ;
- for the edge set $\tilde{D}$ of the subgraph $\tilde{G}$ it holds that |B|+|D~|<(|B|+12) .

Theorem 4.15. Let G=(V∪H,D) be a factor analysis graph and suppose that the tuple $(B,S)\in 2^{V}\times 2^{H}$ satisfies the local BB-criterion. If all nodes ℓ∈S are generically sign-identifiable, then all nodes h∈jpa(B)⧵S are generically sign-identifiable.

Similar to M-identifiability, Theorem 4.15 allows us to recursively certify generic sign-identifiability of a factor analysis graph by checking whether all nodes h∈H are generically sign-identifiable (recall Lemma 2.4).

**Figure 8:**
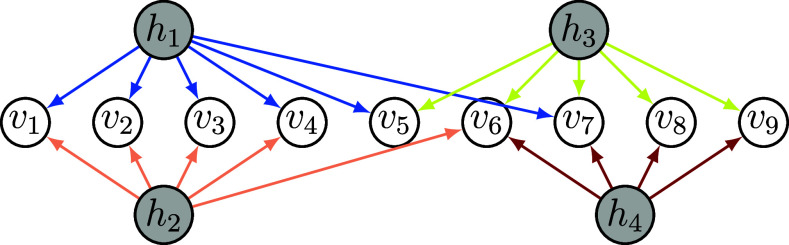
Graph that is certified to be generically sign-identifiable by Theorem 4.15. Figure 8 long description.

Example 4.16. We can use Theorem 4.15 to recursively certify generic sign-identifiability of all latent nodes of the graph displayed in Figure 8.

1. Take $B=\{v_{1},\cdots ,v_{5}\}$ and S=\emptyset such that $\text{jpa}(B)⧵S=\{h_{1},h_{2}\}$ . Observe that GB∪(jpa(B)⧵S) is a full-ZUTA graph such that Condition (iii) is satisfied. Note that the unique ZUTA-ordering on jpa(B) is given by $h_{1}{≺}_{\text{ZUTA}}h_{2}$ , i.e., to verify Condition (ii), we proceed according to this ordering on the latent nodes. The only child of $h_{1}$ that is not a member of *B* is $v_{7}$ . Take any ordering ${≺}_{h_{1}}$ on $\text{ch}(h_{1})$ such that $v_{7}$ is the largest node according to ${≺}_{h_{1}}$ . Then, the ordering ${≺}_{h_{1}}$ is a *B*-first-ordering and $v_{1}{≺}_{h_{1}}v_{7}$ . Moreover, observe that $\text{jpa}(\{v_{1},v_{7}\})=\{h_{1}\}$ . Similarly, we can take any ordering ${≺}_{h_{2}}$ on $\text{ch}(h_{2})$ such that $v_{6}$ is the largest node according to ${≺}_{h_{2}}$ . Since $\text{jpa}(\{v_{1},v_{6}\})=\{h_{2}\}$ , we conclude that Condition (ii) is satisfied.
2. Take $U=\{v_{5},\cdots ,v_{9}\}$ and $S=\{h_{1},h_{2}\}$ such that $\text{jpa}(B)⧵S=\{h_{3},h_{4}\}$ . It is easy to verify that Conditions (i) and (iii) are satisfied. Moreover, we have that $\text{ch}(h_{i})⧵B=\backslash emptyset$ for i=3,4 , that is, Condition (ii) is trivially satisfied.

On the other hand, each observed node in the graph in Figure 8 has at least two latent parents. This implies that ZUTA is not satisfied and hence, due to Corollary 4.11, the graph is not M-identifiable.

Next, we show that the recursive application of Theorem 4.15 subsumes BB-identifiability, that is, we show equivalence on full-ZUTA graphs. Crucially, Theorem 4.15 is also able to certify generic sign-identifiability of *sparse* graphs.

Corollary 4.17. A full-ZUTA graph G=(V∪H,D) is BB-identifiable if and only if generic sign-identifiability of *G* can be certified by recursively applying Theorem 4.15.

We obtain our final criterion by combining Theorems 4.8 and 4.15 iteratively in a recursive algorithm. We call a factor analysis graph *extended M-identifiable* if generic sign-identifiability can be certified recursively by Theorems 4.8 and 4.15 for all nodes h∈H . We have already seen in Example 4.16 that extended M-identifiability may also certify generic sign-identifiability of graphs not satisfying ZUTA. We now provide a further example, where we consider a graph that is extended M-identifiable but applying only one of Theorem 4.8 or Theorem 4.15 does not certify generic sign-identifiability.

**Figure 9:**
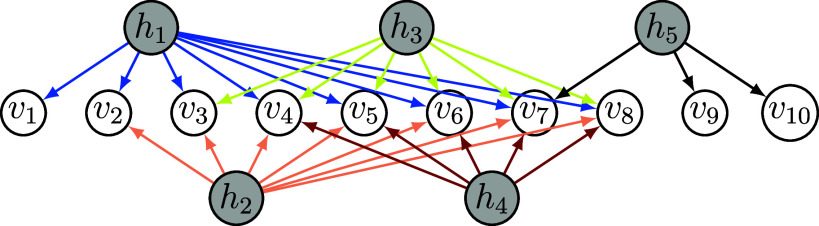
Extended M-identifiable sparse factor analysis graph. Figure 9 long description.

Example 4.18. The factor analysis graph in Figure 9 is an extended M-identifiable. To see this, we recursively check all latent nodes $H=\{h_{1},\cdots ,h_{5}\}$ .

1. The tuple (v,W,U,S) with $v=v_{10}$ , S=\emptyset , $U=\{v_{7}\},$ and $W=\{v_{9}\}$ satisfies the matching criterion with respect to $h_{5}$ . Conditions (i) and (ii) are easily checked, and for Condition (iii), an intersection-free matching is given by $v_{7}\leftarrow h_{5}\to v_{9}$ . To verify Condition (iv), note that $\text{pa}(\{v\}\cup U)\cap \text{pa}(\{v\}\cup W)=\{h_{5}\}$ , which implies that there cannot exist an intersection-free matching of {v}∪U) and {v}∪W .
2. The tuple (B,S) with $B=\{v_{1},\cdots ,v_{8}\}$ and $S=\{h_{5}\}$ satisfies the local BB-criterion and it holds that $\text{jpa}(B)⧵S=H⧵h_{5}$ . The induced subgraph GB∪jpa(B)⧵S) is a full-ZUTA graph on eight observed nodes and four latent nodes for which Condition (iii) holds. Since $\text{ch}(h_{i})⧵B=\backslash emptyset$ for i=1,⋯,4 , Condition (ii) is trivially satisfied.

**Figure 10:**
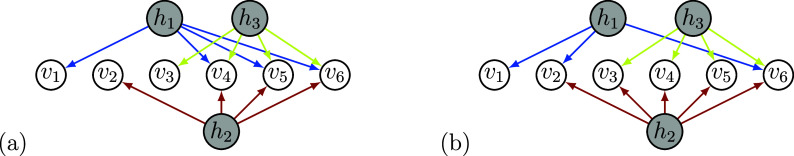
Two generically sign-identifiable graphs that are not extended M-identifiable. Figure 10 long description.

Finally, we emphasize that extended M-identifiability is only sufficient for generic sign-identifiability. Both graphs in Figure 10 can be shown to be generically sign-identifiable via techniques from computational algebra but are not extended M-identifiable.

## 5 Computation

M-identifiability and extended M-identifiability are decidable in polynomial time under certain bounds on the sizes of the subsets involved. In Appendix B of the Supplementary Material, we detail efficient algorithms that are sound and complete.

Theorem 5.1. M-identifiability of a factor analysis graph G=(V∪H,D) is decidable in time $O(|H{|}^{2}|V{|}^{k+1}{\left(|V|+|H|\right)}^{3})$ if we only allow sets *W* with |W|≤k in the matching criterion.

Proof. See Theorem B.4 and Algorithm 2 in the Supplementary Material.

Theorem 5.2. Extended M-identifiability of a factor analysis graph G=(V∪H,D) is decidable in time $O(|H{|}^{2}|V{|}^{\max_{k},l\}+1}{\left(|V|+|H|\right)}^{3})$ if we only allow sets *W* with |W|≤k in the matching criterion and only sets *B* with |B|≤ℓ in the local BB-criterion.

Proof. See Theorem B.8 and Algorithm 6 in the Supplementary Material.

If we allow the cardinality of the sets to be unbounded, then the algorithms we propose in the Supplementary Material search over an exponentially large space and, thus, may take exponential time in the number of nodes. We conjecture that one cannot do significantly better.

Conjecture 5.3. Deciding M-identifiability and extended M-identifiability both is NP-complete.

Remark 5.4. In practice, if there are no restrictions in terms of computational time, then we can allow sets *W* and *B* of arbitrary size. In this case, Algorithm 6 in the Supplementary Material is sound and complete for deciding extended M-identifiability of a latent-factor graph. That is, it returns “yes” if and only if the input graph is extended M-identifiable (see Theorem B.8 in the Supplementary Material). However, the run-time of the unconstrained algorithm is exponential in the number of nodes of the graph. Note that allowing sets of *W* and *B* of unconstrained size is equivalent to choosing k=|H| and ℓ=|V| since |W|≤|H| and |B|≤|V| according to the definitions of the matching criterion and the local BB-criterion. Choosing smaller maximal sizes *k* and ℓ can be useful in practice when attempting to verify generic sign-identifiability of large graphs, where the unconstrained version of Algorithm 6 in the Supplementary Material does not terminate in a reasonable amount of time. With k<|H| and ℓ<|V| , Algorithm 6 in the Supplementary Material is sound but not complete. That is, if the algorithm returns “yes” with k<|H| and ℓ<|V| , then extended M-identifiability holds, which in turn implies generic sign-identifiability. In this case, during the recursive computations, every tuple certified to satisfy the matching criterion fulfills |W|≤k , and every tuple certified to satisfy the local BB-criterion fulfills |B|≤ℓ . However, if the algorithm returns “no” with k<|H| and ℓ<|V| , then we remain inconclusive whether the input graph is extended M-identifiable.

Remark 5.5. Hosszejni and Frühwirth-Schnatter (2026) provide an efficient method to check AR-identifiability in polynomial time by computing maximal matchings in a bipartite graph. As explained in Remark 4.6, their approach is infeasible for checking our matching criterion, as it is a local version of AR-identifiability. The reason why the matching criterion is significantly more computationally intensive is as follows. Recall from Corollary 4.4 that a factor analysis graph that satisfies ZUTA is AR-identifiable if and only if, for all v∈V , there exist two disjoint sets W,U⊆V⧵{v} with |W|=|U|=|H| and an intersection-free matching between *W* and *U*. The crucial difference to Condition (iii) in the matching criterion is that it is already known a priori that the intersection-free matching will involve all nodes h∈H . Checking Condition (iii) in the matching criterion can be seen as checking AR-identifiability locally for every possible subset of latent variables H⊆H . Moreover, the matching criterion needs the additional Condition (iv) to avoid an intersection-free matching between {v}∪W and {v}∪U . This is not needed for AR-identifiability because the existence of such a matching is impossible if |W|=|U|=|H| .

## 6 Numerical experiments

In this section, we first conduct simulations that demonstrate the performance of our criteria. Then, we exemplify how our identifiability criteria are also useful in exploratory factor analysis.

**Table 1:** Counts of unlabeled sparse factor graphs satisfying ZUTA with at most |V|=7 observed nodes and |H|=3 latent nodes Table 1 long description.

No. of edges	ZUTA	gen. sign-id	AR-id	M-id	Ext. M-id
1	1	0	0	0	0
2	2	0	0	0	0
3	4	1	1	1	1
4	7	1	1	1	1
5	14	1	1	1	1
6	25	3	3	3	3
7	41	4	4	4	4
8	56	6	6	6	6
9	73	8	7	8	8
10	77	11	9	11	11
11	79	23	16	23	23
12	67	31	23	29	29
13	54	33	29	33	33
14	31	23	21	23	23
15	18	16	16	16	16
16	8	8	8	8	8
17	4	4	4	4	4
18	1	1	1	1	1
Total	562	174	150	172	172

### 6.1 Simulations

In our simulations, we compare different criteria for generic sign-identifiability. We treat graphs as unlabeled, that is, we count isomorphism classes of graphs. Two factor analysis graphs G=(V∪H,D) and G'=(V∪H,D') on the same set of nodes are isomorphic if there is a permutation $\pi_{V}$ on the observed nodes *V* and a permutation $\pi_{H}$ on the latent nodes H such that, for h∈H and v∈V , the edge h→v∈D if and only if $\pi_{H}(h)\to \pi_{V}(v)\in D'$ .

**Table 2:** Counts of unlabeled sparse factor graphs with at most |V|=9 observed nodes and |H|=4 latent nodes Table 2 long description.

No. of edges	Total	ZUTA	AR-id	M-id	Ext. M-id
1	1	1	0	0	0
2	3	2	0	0	0
3	6	4	1	1	1
4	16	8	1	1	1
5	27	16	1	1	1
6	62	34	3	3	3
7	111	71	4	4	4
8	225	146	9	9	9
9	395	287	14	15	15
10	716	528	21	23	23
11	1,165	922	43	50	50
12	1,880	1,504	81	93	93
13	2,726	2,273	134	167	167
14	3,829	3,192	221	366	368
15	4,890	4,147	404	768	768
16	5,963	4,972	759	1,430	1,435
17	6,599	5,490	1,299	2,187	2,204
18	6,937	5,519	1,927	2,861	2,913
19	6,599	5,047	2,385	3,164	3,273
20	5,963	4,191	2,509	3,037	3,179
21	4,890	3,157	2,231	2,520	2,656
22	3,829	2,139	1,705	1,833	1,913
23	2,726	1,310	1,128	1,177	1,215
24	1,880	710	651	665	683
25	1,165	343	328	331	344
26	716	153	151	151	155
27	395	64	64	64	65
28	225	22	22	22	22
29	111	7	7	7	7
30	62	1	1	1	1
> 30	54	0	0	0	0
Total	64,166	46,260	16,104	20,951	21,568

In our first experimental setup, we consider factor analysis graphs with a small number of observed and latent nodes where generic sign-identifiability can be fully solved by methods from computational algebraic geometry, as we discuss in Appendix D of the Supplementary Material. Table 1 lists counts of all factor analysis graphs with up to three latent nodes and seven observed nodes that satisfy ZUTA. We count how many of the graphs are AR-identifiable, M-identifiable, and extended M-identifiable. For deciding AR-identifiability, we use the algorithm and code provided by Hosszejni and Frühwirth-Schnatter (2026). Note that our criteria are very effective since we only fail to certify the generic sign-identifiability of two graphs. Those are exactly the graphs displayed in Figure 10. Table 1 also illustrates that M-identifiability subsumes AR-identifiability as we have shown in Corollary 4.10. On the other hand, M-identifiability and extended M-identifiability coincide on the considered set of small graphs. This is as expected since the smallest graph, where BB-identifiability holds but M-identifiability does not, has eight observed nodes and four latent nodes.

Our second experimental setup considers all factor analysis graphs with up to four latent nodes and nine observed nodes, and also includes graphs that do not satisfy ZUTA. Table 2 shows that the gap between AR-identifiability and M-identifiability increases. Moreover, extended M-identifiability indeed becomes effective since there are 617 graphs that are extended M-identifiable but not M-identifiable. Recall also that graphs not satisfying ZUTA might be extended M-identifiable but they are never M-identifiable nor AR-identifiable. For computational reasons, we are not able to fully solve generic sign-identifiability by methods from computational algebraic geometry for all graphs considered in Table 2.

Next, to demonstrate that checking extended M-identifiability is feasible on larger graphs with more observed and latent nodes, we randomly generate graphs on 25 observed nodes and 10 latent nodes. We draw the graphs from an Erdös–Renyi model with edge probabilities 0.2 , 0.25 , 0.3 , 0.35 , 0.4 , and 0.45 , where we fix the upper triangle of the adjacency matrix to zero to increase the probability of satisfying ZUTA. Moreover, we only consider graphs with at most 10 children per latent node such that the maximal cardinality of a set *B* satisfying the local BB-criterion is at most 10 . For each edge probability, we sample 5,000 graphs and check whether they are extended M-identifiable. When searching for sets that satisfy the matching criterion, we bound the size of set *W* by k=4 . The number of graphs that were extended M-identifiable is reported in Table 3. Recall that only graphs with at least three children per latent node can be extended M-identifiable. For low edge probabilities, the likelihood is high that this is not satisfied. As expected, the fraction of extended M-identifiable graphs increases with increasing edge probabilities. However, at a certain density level, we would expect that fewer graphs are extended M-identifiable, which we can already see for the edge probability p=0.45 .

**Table 3:** Status of extended M-identifiability for 5,000 randomly generated sparse factor graphs with different edge probabilities with |W|≤k for k=4 Table 3 long description.

Edge probability	0.2	0.25	0.3	0.35	0.4	0.45
Ext. M-identifiable	510	1,784	3,234	4,184	4,536	4,498
Percentage	10.2	35.7	64.7	83.7	90.7	90.0

### 6.2 Application to exploratory factor analysis

In this section, we discuss how our identifiability criteria are also useful in exploratory factor analysis. It is a desirable property in exploratory factor analysis to discover a sparse structure that yields interpretable factor loadings. If we apply threshold-based sparse estimation methods, for example, our identifiability criteria can provide guidance in choosing the threshold or tuning parameter such that identifiability is ensured.

To exemplify this in a small case study, we consider the 2018 Populism and Political Parties Expert Survey (POPPA) that measures positions and attitudes of 250 parties on key attributes related to populism, political style, party ideology, and party organization (Meijers & Zaslove, 2020). The data set is obtained from an expert survey in 28 European countries and contains |V|=15 measured variables. After discarding data points with missing values, 220 samples remain. In their analyses of the data, Meijers and Zaslove (2021) also conduct an exploratory factor analysis. While they retain two latent factors in the main manuscript, they also consider five factors in the Supplementary Material. We replicate their study with five factors by first estimating the loading matrix via maximum likelihood and then applying varimax rotation (Kaiser, 1958) using the factanal function in the base library of R (R Core Team, 2025). We then set all loadings smaller than a predefined threshold to zero to obtain a sparse loading matrix Λ . The associated factor analysis graph includes edge h→v∈D if and only if $\lambda_{hv}\neq 0$ . Note that we do not analyze the two-factor model because generic sign-identifiability is readily ensured as long as at least one measurement does not load on both factors, making the five-factor model a more illustrative setting.

In their analysis, Meijers and Zaslove (2021) use a threshold of 0.5 , which yields a very sparse graph with some factors having less than three children, and thus generic sign-identifiability does not hold (recall Remark 2.5). In contrast, we check whether extended M-identifiability holds for graphs obtained from different thresholds. We obtain that extended M-identifiability only holds for thresholds in the interval 0.10,0.14 . Note that ZUTA is not satisfied for the graphs given by all thresholds in this interval, while it is satisfied for all graphs obtained from thresholds ≥ 0.15. In Table 4, we plot the factor loading matrix that we obtain for the threshold 0.10 . Meijers and Zaslove (2021) argue that the first latent factor represents populism since the first five measurements were designed to measure populism and load strongly on it. For more information on the measured variables, we refer to Meijers and Zaslove (2021).

**Table 4:** Factor loading matrix obtained via maximum likelihood estimation and varimax rotation from the POPPA data set Table 4 long description.

	Factor 1	Factor 2	Factor 3	Factor 4	Factor 5
1	0.799	0.230	0.240	0.141	0.138
2	0.647	0.468	0.264	0.375	
3	0.735	0.356	0.286	0.495	
4	0.921			0.147	
5	0.960				
6	− 0.424	0.541			0.331
7	− 0.242	− 0.894	− 0.192		− 0.107
8	− 0.737	− 0.380	− 0.146	0.113	0.149
9	0.357	0.866	0.185	0.114	0.129
10	0.238	0.887	0.202		
11	− 0.172	− 0.861	− 0.212		
12	− 0.843	− 0.265	− 0.263		
13	0.798	0.302	0.145	0.120	0.316
14	− 0.314	− 0.486	− 0.809		
15	0.289	0.422	0.506		0.413

*Note*: Loadings with absolute value < 0.1 are not shown.

Note that our identifiability conditions can also be incorporated in Bayesian sparse factor analysis, where sparse structures are discovered by employing “spike-and-slab” priors on the factor loading matrix (Conti et al., 2014; Frühwirth-Schnatter et al., 2025; Hosszejni & Frühwirth-Schnatter, 2026). In this case, generic sign-identifiability may be imposed as a domain restriction on the parameter space of the prior distribution. In practice, since the posterior distribution is typically obtained via MCMC sampling, this amounts to post-processing posterior draws under the unrestricted prior by discarding draws whose sparsity pattern does not permit generic sign-identifiability.

## 7 Discussion

We introduced a formal graphical framework for studying identifiability in confirmatory factor analysis when the factor loading matrix is sparse. Our main results provide graphical criteria that constitute sufficient conditions for generic sign-identifiability. It is worth mentioning that even if a model is not extended M-identifiable, it may still be possible to prove generic sign-identifiability of certain columns of the factor loading matrix. This is the case if the recursive algorithm stops early declaring only some but not all latent nodes h∈H to be generically sign-identifiable.

Generic sign-identifiability is useful if an interpretation of the latent factors and their effects on the observed variables is desired. Moreover, if a model is identifiable in this sense, then its dimension equals the expected dimension obtained from counting parameters. This is crucial information for goodness-of-fit tests and model selection procedures.

**Figure 11:**
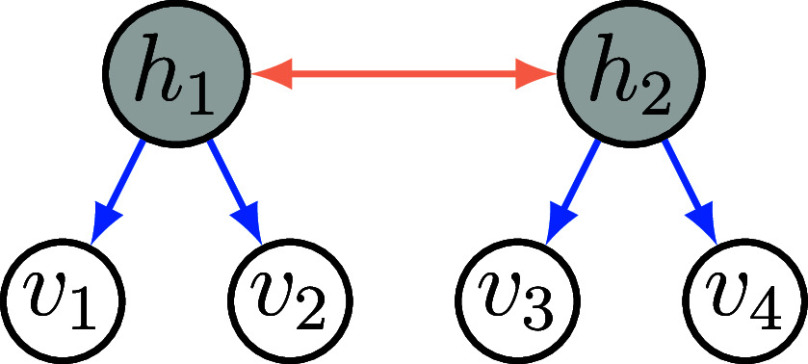
Graph encoding a nonzero correlation between the latent factors.

Our work opens up some natural questions for further studies. For instance, in the generalization of the setup we studied in this article, one may also consider factor analysis models where the factors itself may be correlated. Then, the observed covariance matrix takes the form $\Sigma =\Lambda \Phi \Lambda^{⊤}+\Omega$ for a positive-definite matrix Φ that may also be sparse. For example, consider the graph in Figure 11, where the bidirected edge represents the nonzero correlation between the latent factors. The parameter matrices are then given by

$$\begin{array}{c} \Lambda =\left(\begin{array}{cc} \lambda_{11}\\ 0 & \lambda_{21}\\ 0 & 0\\ \lambda_{32} & 0\\ \lambda_{42} \end{array}\right),\;\Phi =\left(\begin{array}{ccc} 1 & \phi_{12}\phi_{12} & 1 \end{array}\right),\;\text{and}\;\Omega =\left(\begin{array}{ccccccccccccc} \omega_{11} & 0 & 0 & 00 & \omega_{22} & 0 & 00 & 0 & \omega_{33} & 00 & 0 & 0 & \omega_{44} \end{array}\right). \end{array}$$

The observed covariance matrix takes the form

$$\begin{array}{c} \Sigma =(\sigma_{uv})=\left(\begin{array}{cccccc} \omega_{11} & + & \lambda_{11}^{2}\\ \lambda_{11} & \lambda_{21}\\ \lambda_{11} & \phi_{12} & \lambda_{32}\\ \lambda_{11} & \phi_{12} & \lambda_{42} & \lambda_{11} & \lambda_{21}\\ \omega_{22} & + & \lambda_{21}^{2}\\ \lambda_{21} & \phi_{12} & \lambda_{32}\\ \lambda_{21} & \phi_{12} & \lambda_{42} & \lambda_{11} & \phi_{12} & \lambda_{32}\\ \lambda_{21} & \phi_{12} & \lambda_{32}\\ \omega_{33} & + & \lambda_{32}^{2}\\ \lambda_{32} & \lambda_{42} & \lambda_{11} & \phi_{12} & \lambda_{42}\\ \lambda_{21} & \phi_{12} & \lambda_{42}\\ \lambda_{32} & \lambda_{42}\\ \omega_{44} & + & \lambda_{42}^{2} \end{array}\right). \end{array}$$

It is already noted in Bollen (1989, p. 245) that the parameters of this model are identifiable up to sign. Generically, the parameter $\phi_{12}$ is recovered up to sign via the formula

$$\begin{array}{c} \sqrt{\frac{\sigma_{23}\sigma_{14}}{\sigma_{12}\sigma_{34}}}=\sqrt{\frac{\lambda_{21}\phi_{12}\lambda_{32}\lambda_{11}\phi_{12}\lambda_{42}}{\lambda_{11}\lambda_{21}\lambda_{32}\lambda_{42}}}=|\phi_{12}|. \end{array}$$

Given $\left|\phi_{12}\right|$ , we obtain generic identifiability of $\left|\lambda_{11}\right|$ via

$$\begin{array}{c} \sqrt{\frac{\sigma_{13}\sigma_{14}}{\sigma_{34}|\phi_{12}{|}^{2}}}=\sqrt{\frac{\lambda_{11}\phi_{12}\lambda_{32}\lambda_{11}\phi_{12}\lambda_{42}}{\lambda_{32}\lambda_{42}\phi_{12}^{2}}}=|\lambda_{11}|, \end{array}$$

and all other parameters can be recovered similarly. Notably, since each latent node only has two children, generic sign-identifiability is impossible if the correlation $\phi_{12}$ is zero, as we have also seen in Remark 2.5. We believe that our work provides tools for future work on deriving conditions for generic sign-identifiability in models with dependent factors.

Another interesting future research direction is to study structure identifiability, a topic of interest in exploratory factor analysis. Structure identifiability refers to studying whether knowledge of the covariance matrix allows one to uniquely recover an unknown underlying graph. For model selection methods that return sparse factor loading matrices, one might then derive guarantees for recovering a most parsimonious true graph.

## Supporting information

10.1017/psy.2026.10079.sm001 Sturma et al. supplementary material Sturma et al. supplementary material

## Data Availability

An implementation of the algorithms and code for reproducing the experiments is available at https://github.com/MiriamKranzlmueller/id-factor-analysis.

## Supplementary material

The supplementary material for this article can be found at https://doi.org/10.1017/psy.2026.10079.

## Figure 1 Long description

Starting at the top, three shaded nodes labeled h sub 1, h sub 2, and h sub 3 are positioned above six unshaded nodes labeled v sub 1 through v sub 6. H sub 1 connects downward with blue arrows to v sub 1, v sub 2, and v sub 3. H sub 2, located below h sub 1, connects upward with orange arrows to v sub 3, v sub 4, and v sub 5. H sub 3, at the top right, connects downward with green arrows to v sub 4, v sub 5, and v sub 6. Each arrow indicates a directed relationship from a hidden node to a visible node, encoding the sparsity structure of the factor analysis model.

Navigate back to Figure 1.

## Figure 2 Long description

Panel a contains three gray nodes labeled h sub 1, h sub 2, and h sub 3 at the top and bottom, with five white nodes labeled v sub 1 through v sub 5 in a row below. Blue arrows extend from h sub 1 to v sub 1, v sub 2, v sub 3, and v sub 4. Green arrows extend from h sub 1 to v sub 2 and from h sub 3 to v sub 5. Orange arrows extend from h sub 2 to v sub 1, v sub 3, and v sub 5. Panel b has the same node arrangement. Blue arrows from h sub 1 point to v sub 1, v sub 2, v sub 3, and v sub 4. Green arrows from h sub 1 point to v sub 2 and from h sub 3 to v sub 5. Orange arrows from h sub 2 point to v sub 1, v sub 3, v sub 4, and v sub 5. The difference is that in panel b, h sub 2 connects to v sub 4, which is absent in panel a.

Navigate back to Figure 2.

## Figure 4 Long description

Panel a on the left shows three hidden nodes labeled h sub 1, h sub 2, and h sub 3 at the top, and six visible nodes labeled v sub 1 through v sub 6 at the bottom. Blue arrows connect h sub 1 to v sub 1, v sub 2, v sub 3, v sub 4, v sub 5, and v sub 6. Orange arrows connect h sub 2 to v sub 2, v sub 3, v sub 4, v sub 5, and v sub 6. Green arrows connect h sub 3 to v sub 3, v sub 4, v sub 5, and v sub 6. Panel b on the right is similar but includes an additional visible node, v sub 7, at the far right. Blue arrows connect h sub 1 to v sub 1 through v sub 6. Orange arrows connect h sub 2 to v sub 2 through v sub 7. Green arrows connect h sub 3 to v sub 3 through v sub 7. All arrows point downward from hidden nodes to visible nodes.

Navigate back to Figure 4.

## Figure 6 Long description

From left to right, hidden nodes h sub 1, h sub 2, h sub 3, and h sub 4 are shaded and positioned above visible nodes v sub 1 through v sub 9. h sub 1 connects to v sub 1, v sub 2, v sub 3, v sub 4, and v sub 5 with blue arrows. h sub 2 connects to v sub 3, v sub 4, and v sub 5 with orange arrows. h sub 3 connects to v sub 4 and v sub 5 with green arrows. h sub 4 connects to v sub 5, v sub 6, v sub 7, v sub 8, and v sub 9 with red arrows. Each arrow represents a directed sparse factor relationship from hidden to visible nodes.

Navigate back to Figure 6.

## Figure 8 Long description

Starting from the left, h sub 1 is at the top and h sub 2 is below. h sub 1 connects via blue arrows to v sub 1, v sub 2, v sub 3, v sub 4, and v sub 5. h sub 2 connects via orange arrows to v sub 1, v sub 2, v sub 3, v sub 4, v sub 5, and v sub 6. In the center, v sub 1 to v sub 6 are arranged horizontally. Moving right, h sub 3 is above h sub 4. h sub 3 connects via green arrows to v sub 6, v sub 7, v sub 8, and v sub 9. h sub 4 connects via red arrows to v sub 7, v sub 8, and v sub 9. All h nodes are shaded gray, v nodes are white, and arrows are colored to distinguish connection types.

Navigate back to Figure 8.

## Figure 9 Long description

At the top row are five shaded hidden nodes labeled h sub 1, h sub 2, h sub 3, h sub 4, and h sub 5, arranged left to right. Below them are ten unshaded visible nodes labeled v sub 1 through v sub 10, also arranged left to right. Arrows indicate directed connections: h sub 1 connects to v sub 1 through v sub 7 with blue arrows. h sub 2 connects to v sub 2 through v sub 7 with orange arrows. h sub 3 connects to v sub 4 through v sub 7 with green arrows. h sub 4 connects to v sub 5 through v sub 7 with red arrows. h sub 5 connects to v sub 7, v sub 8, v sub 9, and v sub 10 with black arrows. Each arrow points downward from a hidden node to its respective visible nodes, illustrating sparse factor relationships.

Navigate back to Figure 9.

## Figure 10 Long description

Panel a on the left displays h sub 1, h sub 2, and h sub 3 at the top, with v sub 1 through v sub 6 below. Blue arrows from h sub 1 point to v sub 1, v sub 2, v sub 4, and v sub 6. Orange arrows from h sub 2 point to v sub 2, v sub 4, and v sub 6. Green arrows from h sub 3 point to v sub 3, v sub 4, v sub 5, and v sub 6. Panel b on the right shows the same node arrangement. Blue arrows from h sub 1 point to v sub 1, v sub 2, v sub 4, and v sub 6. Orange arrows from h sub 2 point to v sub 2, v sub 4, v sub 5, and v sub 6. Green arrows from h sub 3 point to v sub 3, v sub 4, v sub 5, and v sub 6. The main difference is that in panel b, h sub 2 has an additional arrow to v sub 5 compared to panel a.

Navigate back to Figure 10.

## Table 1 Long description

The table has six columns. The first column lists the number of edges, ranging from 1 to 18, plus a total row. The next five columns are labeled ZUTA, gen. sign-id, AR-id, M-id, and Ext. M-id. For each row, the counts are as follows: for 1 edge, ZUTA is 1, all others are 0; for 2 edges, ZUTA is 2, all others are 0; for 3 edges, ZUTA is 4, the rest are 1; for 4 edges, ZUTA is 7, the rest are 1; for 5 edges, ZUTA is 14, the rest are 1; for 6 edges, ZUTA is 25, the rest are 3; for 7 edges, ZUTA is 41, the rest are 4; for 8 edges, ZUTA is 56, the rest are 6; for 9 edges, ZUTA is 73, gen. sign-id is 8, AR-id is 7, M-id and Ext. M-id are 8; for 10 edges, ZUTA is 77, gen. sign-id is 11, AR-id is 9, M-id and Ext. M-id are 11; for 11 edges, ZUTA is 79, gen. sign-id is 23, AR-id is 16, M-id and Ext. M-id are 23; for 12 edges, ZUTA is 67, gen. sign-id is 31, AR-id is 23, M-id and Ext. M-id are 29; for 13 edges, ZUTA is 54, gen. sign-id is 33, AR-id is 29, M-id and Ext. M-id are 33; for 14 edges, ZUTA is 31, gen. sign-id is 23, AR-id is 21, M-id and Ext. M-id are 23; for 15 edges, ZUTA is 18, gen. sign-id, AR-id, M-id and Ext. M-id are 16; for 16 edges, ZUTA is 8, the rest are 8; for 17 edges, ZUTA is 4, the rest are 4; for 18 edges, ZUTA is 1, the rest are 1. The total row sums to ZUTA 562, gen. sign-id 174, AR-id 150, M-id and Ext. M-id 172.

Navigate back to Table 1.

## Table 2 Long description

The table has six columns. The first column, Number of edges, ranges from 1 to greater than 30, plus a Total row. The next five columns are Total, ZUTA, AR-id, M-id, and Ext. M-id. For 1 edge, the counts are 1 for Total and ZUTA, 0 for the rest. For 2 edges, 3 for Total, 2 for ZUTA, 0 for others. For 3 edges, 6 Total, 4 ZUTA, 1 each for AR-id, M-id, Ext. M-id. For 4 edges, 16 Total, 8 ZUTA, 1 each for AR-id, M-id, Ext. M-id. For 5 edges, 27 Total, 16 ZUTA, 1 each for AR-id, M-id, Ext. M-id. For 6 edges, 62 Total, 34 ZUTA, 3 each for AR-id, M-id, Ext. M-id. For 7 edges, 111 Total, 71 ZUTA, 4 each for AR-id, M-id, Ext. M-id. For 8 edges, 225 Total, 146 ZUTA, 9 each for AR-id, M-id, Ext. M-id. For 9 edges, 395 Total, 287 ZUTA, 14 AR-id, 15 each for M-id and Ext. M-id. For 10 edges, 716 Total, 528 ZUTA, 21 AR-id, 23 each for M-id and Ext. M-id. For 11 edges, 1,165 Total, 922 ZUTA, 43 AR-id, 50 each for M-id and Ext. M-id. For 12 edges, 1,880 Total, 1,504 ZUTA, 81 AR-id, 93 each for M-id and Ext. M-id. For 13 edges, 2,726 Total, 2,273 ZUTA, 134 AR-id, 167 each for M-id and Ext. M-id. For 14 edges, 3,829 Total, 3,192 ZUTA, 221 AR-id, 366 M-id, 368 Ext. M-id. For 15 edges, 4,890 Total, 4,147 ZUTA, 404 AR-id, 768 each for M-id and Ext. M-id. For 16 edges, 5,963 Total, 4,972 ZUTA, 759 AR-id, 1,430 M-id, 1,435 Ext. M-id. For 17 edges, 6,599 Total, 5,490 ZUTA, 1,299 AR-id, 2,187 M-id, 2,204 Ext. M-id. For 18 edges, 6,937 Total, 5,519 ZUTA, 1,927 AR-id, 2,861 M-id, 2,913 Ext. M-id. For 19 edges, 6,599 Total, 5,047 ZUTA, 2,385 AR-id, 3,164 M-id, 3,273 Ext. M-id. For 20 edges, 5,963 Total, 4,191 ZUTA, 2,509 AR-id, 3,037 M-id, 3,179 Ext. M-id. For 21 edges, 4,890 Total, 3,157 ZUTA, 2,231 AR-id, 2,520 M-id, 2,656 Ext. M-id. For 22 edges, 3,829 Total, 2,139 ZUTA, 1,705 AR-id, 1,833 M-id, 1,913 Ext. M-id. For 23 edges, 2,726 Total, 1,310 ZUTA, 1,128 AR-id, 1,177 M-id, 1,215 Ext. M-id. For 24 edges, 1,880 Total, 710 ZUTA, 651 AR-id, 665 M-id, 683 Ext. M-id. For 25 edges, 1,165 Total, 343 ZUTA, 328 AR-id, 331 M-id, 344 Ext. M-id. For 26 edges, 716 Total, 153 ZUTA, 151 AR-id, 151 M-id, 155 Ext. M-id. For 27 edges, 395 Total, 64 ZUTA, 64 AR-id, 64 M-id, 65 Ext. M-id. For 28 edges, 225 Total, 22 ZUTA, 22 AR-id, 22 M-id, 22 Ext. M-id. For 29 edges, 111 Total, 7 ZUTA, 7 AR-id, 7 M-id, 7 Ext. M-id. For 30 edges, 62 Total, 1 ZUTA, 1 AR-id, 1 M-id, 1 Ext. M-id. For more than 30 edges, 54 Total, 0 for all others. The final row sums to 64,166 Total, 46,260 ZUTA, 16,104 AR-id, 20,951 M-id, and 21,568 Ext. M-id.

Navigate back to Table 2.

## Table 3 Long description

The table has six columns for edge probabilities: 0.2, 0.25, 0.3, 0.35, 0.4, and 0.45. For each probability, the first row lists the count of extended M-identifiable graphs out of 5,000: 510 at 0.2, 1,784 at 0.25, 3,234 at 0.3, 4,184 at 0.35, 4,536 at 0.4, and 4,498 at 0.45. The second row gives the corresponding percentages: 10.2 percent, 35.7 percent, 64.7 percent, 83.7 percent, 90.7 percent, and 90.0 percent. The data shows a monotonic increase in both count and percentage of extended M-identifiable graphs as edge probability increases, peaking at 0.4 before a slight decrease at 0.45.

Navigate back to Table 3.

## Table 4 Long description

The table presents a factor loading matrix with 15 rows labeled 1 to 15 and five columns labeled Factor 1 through Factor 5. For each item, loadings are listed under each factor, with some cells blank if the absolute value is less than 0.1. Negative values are indicated with a minus sign. Row 1: 0.799 for Factor 1, 0.230 for Factor 2, 0.240 for Factor 3, 0.141 for Factor 4, 0.138 for Factor 5. Row 2: 0.647 for Factor 1, 0.468 for Factor 2, 0.264 for Factor 3, 0.375 for Factor 4. Row 3: 0.735 for Factor 1, 0.356 for Factor 2, 0.286 for Factor 3, 0.495 for Factor 4. Row 4: 0.921 for Factor 1, 0.147 for Factor 4. Row 5: 0.960 for Factor 1. Row 6: minus 0.424 for Factor 1, 0.541 for Factor 2, 0.331 for Factor 5. Row 7: minus 0.242 for Factor 1, minus 0.894 for Factor 2, minus 0.192 for Factor 3, minus 0.107 for Factor 5. Row 8: minus 0.737 for Factor 1, minus 0.380 for Factor 2, minus 0.146 for Factor 3, 0.113 for Factor 4, 0.149 for Factor 5. Row 9: 0.357 for Factor 1, 0.866 for Factor 2, 0.185 for Factor 3, 0.114 for Factor 4, 0.129 for Factor 5. Row 10: 0.238 for Factor 1, 0.887 for Factor 2, 0.202 for Factor 3. Row 11: minus 0.172 for Factor 1, minus 0.861 for Factor 2, minus 0.212 for Factor 3. Row 12: minus 0.843 for Factor 1, minus 0.265 for Factor 2, minus 0.263 for Factor 3. Row 13: 0.798 for Factor 1, 0.302 for Factor 2, 0.145 for Factor 3, 0.120 for Factor 4, 0.316 for Factor 5. Row 14: minus 0.314 for Factor 1, minus 0.486 for Factor 2, minus 0.809 for Factor 3. Row 15: 0.289 for Factor 1, 0.422 for Factor 2, 0.506 for Factor 3, 0.413 for Factor 5. The note states that loadings with absolute value less than 0.1 are not shown.

Navigate back to Table 4.
