# Supplementary material for: Matching Criterion for Identifiability in Sparse Factor Analysis
Source: Psychometrika. 2026 Jan 20;91(2):536–55. doi: 10.1017/psy.2026.10079 (PMC13294627; doi:10.1017/psy.2026.10079)
Supplement: Sturma et al. supplementary material [file S0033312326100799sup001.pdf]

Appendix

A Additional Lemmas for Full Factor Models

We relate the existing literature on identifiability of full factor analysis models to our setup that also allows for sparse factor models. To formally define generic identifiability of the diagonal matrix Ω, we use the same terminology as Bekker and ten Berge (1997). Let π_{diag} be the projection of the parameters space Θ_G to the parameters corresponding to the diagonal matrix, that is,

$$\begin{aligned} \pi_{\text{diag}} : \Theta_G &\longrightarrow \mathbb{R}^{|V|}_{>0} \\ (\Omega, \Lambda) &\longmapsto \Omega. \end{aligned} \tag{3}$$

Definition A.1. A factor analysis graph $G = (V \cup \mathcal{H}, D)$ is said to be *generically globally identifiable* if $\pi_{\text{diag}}(\mathcal{F}_G(\Omega, \Lambda)) = \{\Omega\}$ for almost all $(\Omega, \Lambda) \in \Theta_G$.

We have the following lemma.

Lemma A.2. *Let $G = (V \cup \mathcal{H}, D)$ be a factor analysis graph such that ZUTA is satisfied. Then G is generically globally identifiable if and only if G is generically sign-identifiable.*

Proof. If G is generically sign-identifiable, then by definition it is generically globally identifiable. For the other direction, let G be generically globally identifiable. Fix a generically chosen parameter tuple $(\Omega, \Lambda) \in \Theta_G$ and consider a tuple $(\widetilde{\Omega}, \widetilde{\Lambda}) \in \mathcal{F}_G(\Omega, \Lambda)$ in the fiber of (Ω, Λ). It holds that $\Lambda\Lambda^\top + \Omega = \widetilde{\Lambda}\widetilde{\Lambda}^\top + \widetilde{\Omega}$. Since G is generically globally identifiable, it follows that $\Omega = \widetilde{\Omega}$, and therefore $\Lambda\Lambda^\top = \widetilde{\Lambda}\widetilde{\Lambda}^\top$. We assume w.l.o.g. that each latent node has at least one child since otherwise the node is trivially generically-sign identifiable. Since ZUTA is satisfied it holds that Λ generically has full column rank. By multiplying

from the right with the Moore-Penrose pseudoinverse of $\widetilde{\Lambda}^\top$, it must hold that $\widetilde{\Lambda} = \Lambda Q$ for some invertible matrix $Q \in \mathbb{R}^{|\mathcal{H}| \times |\mathcal{H}|}$. Moreover, this matrix Q has to be orthogonal, since otherwise the equality $\Lambda\Lambda^\top = \widetilde{\Lambda}\widetilde{\Lambda}^\top = \Lambda Q Q^\top \Lambda^\top$ does not hold; to see this multiply with the pseudoinverses of Λ and $\Lambda^\top$ again.

For finishing the proof, we claim that $\Lambda Q \in \mathbb{R}^D$ for an orthogonal matrix Q if and only if Q is diagonal with entries in $\{\pm 1\}$. Assume w.l.o.g. that the latent nodes are ordered by the ZUTA-ordering. Then, there also exists an ordering of the observed nodes such that Λ contains a lower triangular submatrix $\Lambda_{U,\mathcal{H}}$ with no zeros on the diagonal for a subset $U \subseteq V$ with $|U| = |\mathcal{H}|$. We can choose the subset U such that, for generically chosen Λ, the lower triangular matrix $\Lambda_{U,\mathcal{H}}$ is nonsingular. Observe that $\Lambda Q \in \mathbb{R}^D$ implies that the matrix $T := \Lambda_{U,\mathcal{H}} Q$ also has to be lower triangular. Hence, $Q = \Lambda_{U,\mathcal{H}}^{-1} T$ is also lower triangular. But then $Q^{-1} = Q^\top$ has to be lower triangular as well, which implies that Q is diagonal. Finally, note that $I = Q^\top Q$ which implies that the squared diagonal entries of Q are all equal to one. □

Existing literature mainly focused on full factor models, which correspond to the following graphs.

Definition A.3. A *full* factor analysis graph is a factor analysis graph $G = (V \cup \mathcal{H}, D)$ with $D = \mathcal{H} \times V$.

Now, we show that every full factor analysis graph is in a one-to-one relation to the full-ZUTA graph on the same set of nodes.

Lemma A.4. *Let $G = (V \cup \mathcal{H}, \mathcal{H} \times V)$ be a full factor analysis graph and consider the corresponding full-ZUTA graph $G' = (V \cup \mathcal{H}, D)$ on the same set of nodes. Then $F(G) = F(G')$ and, moreover, G is generically globally identifiable if and only if G' is generically sign-identifiable.*

Proof. The inclusion $F(G') \subseteq F(G)$ is trivial since the edges of G' are a subset of the edges of G. For the other inclusion, consider a point $\Sigma \in F(G)$ in the full factor analysis model. Then $\Sigma = \Lambda\Lambda^\top + \Omega$ for some parameters $(\Omega, \Lambda) \in \Theta_G$. As in the QR-decomposition, there is an orthogonal matrix Q such that ΛQ has a zero upper triangle, that is, $\Lambda Q \in \mathbb{R}^D$ where D is the edge set of the full-ZUTA graph G'. Since $\Lambda Q Q^\top \Lambda^\top + \Omega = \Lambda\Lambda^\top + \Omega = \Sigma$, we conclude that $\Sigma \in F(G')$.

For the second statement, observe that G is generically globally identifiable if and only if G' is generically globally identifiable. Since G' satisfies ZUTA, it follows by Lemma A.2 that G' is generically globally identifiable if and only if G' generically sign-identifiable. $\square$

B Algorithms

In this section, we propose efficient algorithms for deciding whether a sparse factor analysis graph is M-identifiable or extended M-identifiable.

B.1 Deciding M-identifiability

Let $G = (V \cup \mathcal{H}, D)$ be a factor analysis graph and fix a node $h \in \mathcal{H}$. When recursively checking M-identifiability, the next lemma verifies that we can always take the set $S \subseteq \mathcal{H} \setminus \{h\}$ to be the set of *all* previously solved nodes, that is, all latent nodes that are already known to be generically sign-identifiable.

Lemma B.1. *Fix a latent node $h \in \mathcal{H}$ in a factor analysis graph $G = (V \cup \mathcal{H}, D)$. Let $\widetilde{S} \subseteq \mathcal{H} \setminus \{h\}$, and suppose that $(v, W, U, S) \in V \times 2^V \times 2^V \times 2^{\mathcal{H}\setminus\{h\}}$ with $S \subseteq \widetilde{S}$ satisfies the matching criterion with respect to h. Then there are $\widetilde{W}, \widetilde{U} \subseteq V$ such that the tuple $(v, \widetilde{W}, \widetilde{U}, \widetilde{S})$ also satisfies the matching criterion with respect to h.*

Proof. Define $W = \{w_1, \ldots, w_k\}$ and $U = \{u_1, \ldots, u_k\}$ and let $\Pi = \{\pi_1, \ldots, \pi_k\}$ be an intersection-free matching of W and U such that π_i is given by $w_i \leftarrow h_i \rightarrow u_i$. Since Π avoids S, each latent node h_i that appears in Π is not in S. If a latent node h_i is in $\widetilde{S}$, we remove w_i from W and u_i from U. This defines $\widetilde{W}$ and $\widetilde{U}$ as subsets of W and U respectively. Now, we check that the tuple $(v, \widetilde{W}, \widetilde{U}, \widetilde{S})$ satisfies conditions (i) - (iv) of the matching criterion; recall Definition 4.7.

To check condition (i), recall that $h \notin \widetilde{S}$ and $S \subseteq \widetilde{S}$. Hence, it directly follows from $\text{pa}(v) \setminus S = \{h\}$ that $\text{pa}(v) \setminus \widetilde{S} = \{h\}$ also holds. Moreover, we have that $v \notin \widetilde{W} \cup \widetilde{U}$ since $v \notin W \cup U$.

To check condition (ii), first note that $\widetilde{W}$ and $\widetilde{U}$ are subsets of the disjoint sets W and U. Thus, the sets $\widetilde{W}$ and $\widetilde{U}$ are also disjoint. To see that $\widetilde{W}$ and $\widetilde{U}$ are nonempty, observe that by the definition of the matching criterion, h is equal to one of the latent nodes $h_1, \ldots, h_k$ appearing in Π, say $h = h_j$ for some $j \in [k]$. Since $h \notin \widetilde{S}$, we have not removed w_j and u_j from W and U. Hence, the sets $\widetilde{W}$ and $\widetilde{U}$ are nonempty. Clearly, they also have equal cardinality.

To check condition (iii) note that the paths $\pi_i \in \Pi$ that do not visit a latent node $h_i \in \widetilde{S}$ define a intersection-free matching of $\widetilde{W}$ and $\widetilde{U}$.

Finally, it can be seen by contradiction that condition (iv) is also satisfied. Suppose there is an intersection free matching of $\{v\} \cup \widetilde{W}$ and $\{v\} \cup \widetilde{U}$ that avoids $\widetilde{S}$. Adding the paths $\pi_i : w_i \leftarrow h_i \rightarrow u_i$ with $h_i \in \widetilde{S} \setminus S$ to this matching, gives an intersection-free matching of $\{v\} \cup W$ and $\{v\} \cup U$. This is a contradiction and we conclude that there can not exist an intersection-free matching of $\{v\} \cup \widetilde{W}$ and $\{v\} \cup \widetilde{U}$ that avoids $\widetilde{S}$. □

For a given node $h \in \mathcal{H}$ and a set $S \subseteq \mathcal{H} \setminus \{h\}$, we denote by $\mathtt{M}(G, h, S)$ the decision problem whether there exists a tuple $(v, W, U) \in V \times 2^V \times 2^V$ such that (v, W, U, S) satisfies

the matching criterion with respect to h. To decide $\mathtt{M}(G, h, S)$, we make use of maximum flows in a special flow graph $G_{\text{flow}} = (V_f, D_f)$ from a designated source node $s \in V_f$ to a target node $t \in V_f$. The standard maximum-flow framework is introduced in Cormen, Leiserson, Rivest, and Stein (2009), and a maximum-flow in an acyclic directed graph can be computed in polynomial time with complexity $\mathcal{O}(|V_f|^3)$.

We first address the subproblem of verifying whether a given tuple $(v, W, U, S) \in V \times 2^V \times 2^V \times 2^{\mathcal{H}\setminus\{h\}}$ satisfies the matching criterion with respect to h. Conditions (i)-(ii) in the definition of the matching criterion are easy to check. If we suppose that they are satisfied, then we are able to check whether Condition (iii) holds, i.e., whether there exists an intersection-free matching of W and U that avoids S, by one maximum flow computation on a suitable flow graph $G^{(iii)}_{\text{flow}}(v, W, U, S) = (V_{(iii)}, D_{(iii)})$. The nodes of the flow graph are given by $V_{(iii)} = U \cup W \cup (\mathcal{H} \setminus S) \cup \{s, t\}$, where s is a source node and t is a target node. The set of edges is given by the union

$$
\begin{aligned}
D_{(iii)} = &\{s \to w : w \in W\} \\
&\cup \{w \to h : h \in \mathcal{H} \setminus S, w \in W, h \to w \in D\} \\
&\cup \{h \to u : h \in \mathcal{H} \setminus S, u \in U, h \to u \in D\} \\
&\cup \{u \to t : u \in U\}.
\end{aligned}
$$

We assign to the source node s and the target node t the capacity ∞, while all other nodes have capacity 1. The edges all have capacity ∞. By construction, no flow in $G^{(iii)}_{\text{flow}}(v, W, U, S)$ can exceed $|W| = |U|$ in size, hence one may replace the infinite capacities with $|W| = |U|$ in practice. To check whether Condition (iv) holds, we construct a second flow graph $G^{(iv)}_{\text{flow}}(v, W, U, S) = (V_{(iv)}, D_{(iv)})$ by adding some nodes and edges to the flow graph $G^{(iii)}_{\text{flow}}(v, W, U, S)$. Let v' be a copy of v. Then the graph $G^{(iv)}_{\text{flow}}(v, W, U, S)$

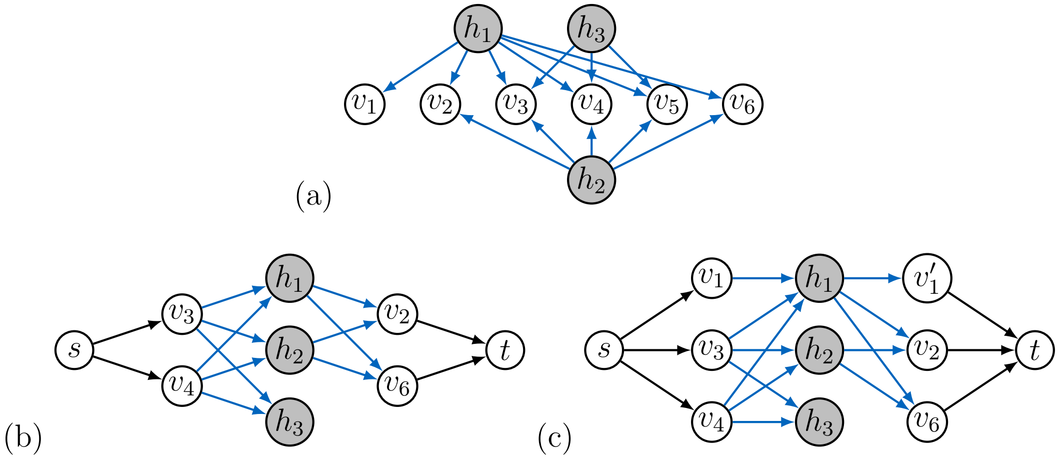

Figure 12: Using maximum flow to verify whether the tuple $(v, W, U, S) = (v_1, \{v_3, v_4\}, \{v_2, v_6\}, \emptyset)$ satisfies Conditions (iii) and (iv) of the matching criterion. (a) The considered sparse factor analysis graph. (b) The flow graph $G^{(iii)}_{\text{flow}}(v, W, U, S)$. (c) The flow graph $G^{(iv)}_{\text{flow}}(v, W, U, S)$.

contains the nodes $V_{(iv)} = V_{(iii)} \cup \{v, v'\}$ and the edges

$$\begin{aligned} D_{(iv)} = D_{(iii)} &\cup \{s \to v\} \\ &\cup \{v \to h : h \in \mathcal{H} \setminus S, h \to v \in D\} \\ &\cup \{h \to v' : h \in \mathcal{H} \setminus S, h \to v' \in D\} \\ &\cup \{v' \to t\}. \end{aligned}$$

Similarly as before, we assign to all edges and to the source node s and the target node t the capacity ∞, while we assign to all other nodes the capacity 1. Since no flow in $G^{(iv)}_{\text{flow}}(v, W, U, S)$ can exceed $|W| + 1$ in size, we replace the infinite capacities with $|W| + 1$ in practice. An example of a both flow graphs is shown in Figure 12.

Let $\texttt{MaxFlow}(G^{(iii)}_{\text{flow}}(v, W, U, S))$ be the maximum flow from s to t in the flow graph $G^{(iii)}_{\text{flow}}(v, W, U, S)$ and let $\texttt{MaxFlow}(G^{(iv)}_{\text{flow}}(v, W, U, S))$ be the maximum flow from s to t in the flow graph $G^{(iv)}_{\text{flow}}(v, W, U, S)$. We have the following result.

Lemma B.2. *Let $G = (V \cup \mathcal{H}, D)$ be a factor analysis graph and fix a latent node $h \in \mathcal{H}$. Suppose that the tuple $(v, W, U, S) \in V \times 2^V \times 2^V \times 2^{\mathcal{H}\setminus\{h\}}$ satisfies Conditions (i) and (ii) of the matching criterion with respect to h. Then, Conditions (iii) and (iv) of the matching criterion are satisfied if and only if* $\texttt{MaxFlow}(G^{(iii)}_{\mathit{flow}}(v, W, U, S)) = |W|$ *and* $\texttt{MaxFlow}(G^{(iv)}_{\mathit{flow}}(v, W, U, S)) = |W|$.

Proof. The proof is along the lines of the proofs of Foygel, Draisma, and Drton (2012, Theorem 6) and Barber et al. (2022, Theorem 5.1). Let $(v, W, U, S) \in V \times 2^V \times 2^V \times 2^{\mathcal{H}\setminus\{h\}}$ be a tuple that satisfies Conditions (i) and (ii) of the matching criterion with respect to h. First, we show that Condition (iii) is satisfied if and only if $\texttt{MaxFlow}(G^{(iii)}_{\text{flow}}(v, W, U, S)) = |W|$. Suppose Condition (iii) is satisfied, i.e., there an intersection-free matching Π of W and U that avoids S. For each path $\pi \in \Pi$ of the form

$$\pi : w \leftarrow h \rightarrow u,$$

with $w \in W$, $u \in U$ and $h \in \mathcal{H} \setminus S$, add a flow of size 1 along the path

$$\widetilde{\pi} : s \rightarrow u \rightarrow h \rightarrow w \rightarrow t$$

in the flow graph $G^{(iii)}_{\text{flow}}$. Let $\widetilde{\Pi}$ be the set of paths that we obtain in the flow graph $G^{(iii)}_{\text{flow}}$. Observe that the total flow size from s to t in the flow graph is equal to $|W| = |U|$. It is left to check that no capacity constraint is exceeded. This is trivial for the infinite edge capacities as well as for the infinite capacities of the source node s and the target node t. Note that all other nodes that appear in some of the paths of the set $\widetilde{\Pi}$ appear exactly once since the original set of paths Π is intersection-free and $W \cap U = \emptyset$.

Now, suppose for the other direction that $\texttt{MaxFlow}(G^{(iii)}_{\text{flow}}(v, W, U, S)) = |W|$. Based on the properties of the max-flow problem with integer capacities (Ford & Fulkerson, 1962), this implies that there are $|W|$ directed path from s to t, each having a flow of size 1. Let

$\widetilde{\Pi}$ be the collection of these paths. Each node different from s and t can appear at most once in the set of paths $\widetilde{\Pi}$. By construction, each path $\widetilde{\pi} \in \widetilde{\Pi}$ has the form

$$\widetilde{\pi} : s \to w \to h \to u \to t,$$

with $w \in W$, $u \in U$ and $h \in \mathcal{H} \setminus S$. This defines the set of paths Π in the factor analysis graph G, where each path $\pi \in \Pi$ is of the form

$$\pi : w \leftarrow h \to u.$$

By construction, Π is an intersection-free matching from W to U since each node other than s or t appears at most once in the set of paths $\widetilde{\Pi}$.

Equivalently, we can see that there is an intersection-free matching of $W \cup \{v\}$ and $U \cup \{v\}$ that avoids S if and only if $\texttt{MaxFlow}(G_{\text{flow}}^{(iv)}(v, W, U, S)) = |W| + 1$. We conclude the proof by noting that it must hold that $\texttt{MaxFlow}(G_{\text{flow}}^{(iv)}(v, W, U, S)) \in \{|W|, |W| + 1\}$ whenever $\texttt{MaxFlow}(G_{\text{flow}}^{(iii)}(v, W, U, S)) = |W|$. □

Lemma B.2 implies that the decision problem $\texttt{M}(G, h, S)$ is in the NP-complexity class. Every candidate tuple (v, W, U) to solve $\texttt{M}(G, h, S)$ can be checked to be a solution in polynomial time by first checking if the tuple satisfies Conditions (i) and (ii) of the matching criterion and then verifying whether $\texttt{MaxFlow}(G_{\text{flow}}^{(iii)}) = |W|$ and $\texttt{MaxFlow}(G_{\text{flow}}^{(iv)}) = |W|$.

We now give an algorithm to decide $\texttt{M}(G, h, S)$ by iterating over all suitable tuples $(v, W, U) \in V \times 2^V \times 2^V$. By applying Lemma B.2, we check for each tuple whether it satisfies the matching criterion with respect to h. The next fact simplifies the search for tuples that satisfy the matching criterion.

Lemma B.3. *Let $G = (V \cup \mathcal{H}, D)$ be a factor analysis graph. If the tuple $(v, W, U, S) \in V \times 2^V \times 2^V \times 2^{\mathcal{H}\setminus\{h\}}$ satisfies the matching criterion with respect to a latent node $h \in \mathcal{H}$, then it holds that $h \in pa(W) \cap pa(U)$.*

Algorithm 1 Deciding $\mathtt{M}(G, h, S)$.

Input: Factor analysis graph $G = (V \cup \mathcal{H}, D)$, a node $h \in \mathcal{H}$, and a set $S \subseteq \mathcal{H} \setminus \{h\}$.

1: **for** $v \in \mathrm{ch}(h)$ such that $\mathrm{pa}(v) \setminus S = \{h\}$ **do**
2:   **for** $W \subseteq V \setminus \{v\}$ such that $h \in \mathrm{pa}(W)$ and $|W| \leq \min\{(|V|-1)/2, |\mathcal{H} \setminus S|\}$ **do**
3:     **for** $U \subseteq \mathrm{ch}(\mathrm{pa}(W) \setminus S) \setminus (\{v\} \cup W)$ such that $h \in \mathrm{pa}(U)$ and $|U| = |W|$ **do**
4:       **if** $\mathtt{MaxFlow}(G_{\text{flow}}^{(iii)}(v, W, U, S)) = |W|$ and $\mathtt{MaxFlow}(G_{\text{flow}}^{(iv)}(v, W, U, S)) = |W|$ **then**
5:         **Output:** "yes".
6:       **end if**
7:     **end for**
8:   **end for**
9: **end for**
10: **Output:** "no".

Proof. Let $h \in \mathcal{H}$ be a latent node and suppose that the tuple $(v, W, U, S) \in V \times 2^V \times 2^V \times 2^{\mathcal{H} \setminus \{h\}}$ satisfies the matching criterion with respect to h. By Condition (iii) of the matching criterion, there exists an intersection-free matching Π of W and U that avoids S. If $h \notin \mathrm{pa}(W)$ or $h \notin \mathrm{pa}(U)$, then h does not appear in Π. Therefore, by adding the path $v \leftarrow h \rightarrow v$ to Π, we obtain an intersection-free matching of $\{v\} \cup W$ and $\{v\} \cup U$ that avoids S. This is a contradiction to Condition (iv) of the matching criterion and we conclude that it must hold that $h \in \mathrm{pa}(W) \cap \mathrm{pa}(U)$. □

Our procedure to decide $\mathtt{M}(G, h, S)$ is formalized in Algorithm 1.

Theorem B.4. *Algorithm 1 is sound and complete for deciding $\mathtt{M}(G, h, S)$. If we only allow sets W with $|W| \leq k$ in line 2, then the algorithm has complexity at most $\mathcal{O}(|V|^{k+1}(|V| + |\mathcal{H}|)^3)$.*

Proof. The algorithm is sound and complete by Lemma B.3 and Lemma B.2. For the complexity, note that we run the "inner" algorithm (lines 2 to 8) at most $|V|$ times. In the inner algorithm we iterate at most over all sets $W \subseteq V$ with cardinality at most k in line

Algorithm 2 Deciding M-identifiability.

Input: Factor analysis graph $G = (V \cup \mathcal{H}, D)$.
Initialize: Solved nodes $S \leftarrow \{h \in \mathcal{H} : \mathrm{ch}(h) = \emptyset\}$.

```
1: repeat
2:    for h ∈ H \ S do
3:       if M(G, h, S) holds then
4:          S ← S ∪ {h}.
5:          break
6:       end if
7:    end for
8: until S = H or no change has occurred in the last iteration.
9: Output: "yes" if S = H, "no" otherwise.
```

s. The number of subsets of W with cardinality at most k is

$$\sum_{i=0}^{k} \binom{|V|}{i} = \mathcal{O}(|V|^k)$$

In line 3 we then iterate at most over all $U \subseteq V$ with $|U| = |W| = k$. Thus, we compute at most $\mathcal{O}(|V|^{k+1})$ maximum flows on acyclic graphs with at most $|V| + |\mathcal{H}| + 3$ nodes. By Cormen et al. (2009, Section 26), each maximum flow computation is of complexity $\mathcal{O}((|V|+|\mathcal{H}|)^3)$, and we conclude that the total complexity of Algorithm 1 is $\mathcal{O}(|V|^{k+1}(|V|+|\mathcal{H}|)^3)$. □

Finally, we provide a procedure for deciding M-identifiability in Algorithm 2, where we iterate over all nodes $h \in \mathcal{H} \setminus S$ and solve $\mathtt{M}(G, h, S)$ in each step. It is easy to see that the algorithm is sound and complete. Under the same constraints as in Theorem B.4, the complexity is at most $\mathcal{O}(|\mathcal{H}|^2|V|^{k+1}(|V| + |\mathcal{H}|)^3)$.

B.2 Deciding Extended M-identifiability

In this section, we provide an algorithm for deciding extended M-identifiability in a factor analysis graph. We start by proposing a procedure to find a tuple that satisfies the local BB-criterion; recall Theorem 4.15. Denote by $\mathtt{L}(G, S)$ the subproblem of deciding whether

there exists a set $B \subseteq V$ such that the tuple (B, S) satisfies the local BB-criterion for a fixed set of latent nodes $S \subseteq \mathcal{H}$. The next lemma verifies that we can always fix the set S to be the set of all nodes already proven to be generically sign-identifiable.

Lemma B.5. *Let $G = (V \cup \mathcal{H}, D)$ be a factor analysis graph and suppose that the tuple $(B, S) \in 2^V \times 2^{\mathcal{H}}$ satisfies the local BB-criterion. Let $h \in jpa(B) \setminus S$ be a latent node. Then there is $\widetilde{B} \in 2^V$ with $|\widetilde{B}| = |B| - 1$ and $jpa(\widetilde{B}) \setminus (S \cup h) = jpa(B) \setminus (S \cup h)$ such that $(\widetilde{B}, S \cup h)$ satisfies the local BB-criterion.*

Proof. Let $p = |B|$ and $m = |\text{jpa}(B) \setminus S|$. Since $G[B \cup (\text{jpa}(B) \setminus S)]$ is a full-ZUTA graph, there is a unique observed node u_ℓ for each $\ell \in \text{jpa}(B) \setminus S$ such that $u_\ell \in \text{ch}(\ell)$ and $u_\ell \notin \bigcup_{k \succ_{\text{ZUTA}} \ell} \text{ch}(k)$. Now, let $h \in \text{jpa}(B) \setminus S$ be a latent node and define $\widetilde{B}$ as

$$\widetilde{B} = B \setminus \{u_h\}.$$

Note that $|\widetilde{B}| = |B| - 1$. We claim that the pair $(\widetilde{B}, S \cup h)$ satisfies the local BB-criterion. However, we first show that $\text{jpa}(\widetilde{B}) \setminus (S \cup h) = \text{jpa}(B) \setminus (S \cup h)$. Since $\widetilde{B} \subseteq B$, one inclusion is clear. For the other inclusion, take a latent node $\ell \in \text{jpa}(B) \setminus (S \cup h)$. Observe that the corresponding node u_ℓ is an element of $\widetilde{B}$. Moreover, since $m < p$ and (B, S) satisfies the local BB-criterion, there is $w \in B$ such that $w \in \text{ch}(k) \setminus \{u_k\}$ for all $k \in \text{jpa}(B) \setminus S$. Hence, it must be the case that $w \in \widetilde{B}$, and since $\ell \in \text{jpa}(u_\ell, w)$, we conclude that $\ell \in \text{jpa}(\widetilde{B}) \setminus (S \cup h)$.

It is easy to see that the pair $(\widetilde{B}, S \cup h)$ satisfies Condition (i) of the local BB-criterion. To check Condition (ii), consider a latent node $\ell \in \text{jpa}(B) \setminus (S \cup h)$. If $\ell \succ_{\text{ZUTA}} h$, then $u_h \notin \text{ch}(\ell)$, and the B-first ordering $\prec_\ell$ on $\text{ch}(\ell)$ is also a $\widetilde{B}$-first ordering on $\text{ch}(\ell)$ that satisfies condition (ii).

Now, consider a latent node $\ell \in \text{jpa}(B) \setminus (S \cup h)$ with $\ell \prec_{\text{ZUTA}} h$. Note that $\prec_\ell$ is a $\widetilde{B}$-first-ordering on $\text{ch}(\ell) \setminus \{u_h\}$. Now, we extend this ordering to an ordering $\widetilde{\prec}_\ell$ on the

whole set of children $\mathrm{ch}(\ell)$. We define it as the block-ordering

$$\widetilde{B} \;\,\widetilde{\prec}_\ell\;\, \{u_h\} \;\,\widetilde{\prec}_\ell\;\, \mathrm{ch}(\ell) \setminus B, \tag{4}$$

where, for two sets A, C, we write $A \,\widetilde{\prec}_\ell\, C$ if $a \,\widetilde{\prec}_\ell\, c$ whenever $a \in A$ and $c \in C$ in the ordering $\widetilde{\prec}_\ell$. Moreover, within each set in (4), the ordering $\widetilde{\prec}_\ell$ coincides with the ordering $\prec_\ell$. Clearly, the ordering in (4) is a $\widetilde{B}$-first ordering on the set of children $\mathrm{ch}(h)$. To show that is satisfies condition (ii) of the local BB-criterion, consider first the node u_h. Take the node u_ℓ and observe that it satisfies $u_\ell \,\widetilde{\prec}_\ell\, u_h$ and $\mathrm{jpa}(\{u_\ell, u_h\}) \setminus (S \cup h) \subseteq \{k \in \mathrm{jpa}(\widetilde{B}) \setminus (S \cup h) : k \preceq_{\mathrm{ZUTA}} \ell\}$. Now, take any other node $v \in \mathrm{ch}(\ell) \setminus B$. Then the existence of a suitable node $u \in \mathrm{ch}(\ell)$ is ensured by the fact that $\widetilde{B} = B \cup \{u_h\}$. Hence $(\widetilde{B}, S \cup h)$ satisfies Condition (ii) the local BB-criterion.

To finish the proof, it remains to show Condition (iii) of the local BB-criterion. Recall that, for $p = |B|$ and $m = |\mathrm{jpa}(B) \setminus S|$, it holds that $p(m+1) - \binom{m}{2} < \binom{p+1}{2}$. Now, let $\widetilde{p} = p - 1$ and $\widetilde{m} = m - 1$, and consider the following chain of equivalent statements:

$$\begin{aligned}
& p(m+1) - \binom{m}{2} < \binom{p+1}{2} \\
\iff\; & pm - m + m + p - \left\{\binom{m-1}{2} + (m-1)\right\} < \binom{p}{2} + p \\
\iff\; & (p-1)m - \binom{m-1}{2} + 1 < \binom{p}{2} \\
\iff\; & \widetilde{p}(\widetilde{m}+1) - \binom{\widetilde{m}}{2} < \binom{\widetilde{p}+1}{2}.
\end{aligned}$$

We conclude that the pair $(\widetilde{B}, S \cup h)$ satisfies the local BB-criterion. □

Next, we provide an algorithm for deciding whether a factor analysis graph is a full-ZUTA graph.

Lemma B.6. *A sparse factor analysis graph $G = (V \cup \mathcal{H}, D)$ is a full-ZUTA graph if and only if Algorithm 3 returns "yes". Moreover, the algorithm has complexity at most $\mathcal{O}(|\mathcal{H}||V|^2)$.*

Algorithm 3 Deciding full-ZUTA.

Input: Factor analysis graph $G = (V \cup \mathcal{H}, D)$ with $|\mathcal{H}| \leq |V|$.
Initialize: $p \leftarrow |V|$ and $m \leftarrow |\mathcal{H}|$.

1: Relabel $\mathcal{H} \leftarrow \{h_1, \ldots, h_m\}$ such that $\mathrm{ch}(h_i) \leq \mathrm{ch}(h_{i+1})$ for all $i \in [m-1]$.
2: **for** $i \in [m]$ **do**
3:     **if** $|\mathrm{ch}(h_i)| \neq p - i + 1$ **then**
4:         **return** "no".
5:     **end if**
6: **end for**
7: **for** $i \in [m-1]$ **do**
8:     $W \leftarrow \mathrm{ch}(h_i) \setminus \mathrm{ch}(h_{i+1})$.
9:     **if** $|W| \neq 1$ **then**
10:         **return** "no".
11:     **else**
12:         $W = \{w\}$.
13:         **for** $j \in \{i+2, \ldots, m\}$ **do**
14:             **if** $w \in \mathrm{ch}(h_j)$ **then**
15:                 **return** "no".
16:             **end if**
17:         **end for**
18:     **end if**
19: **end for**
20: **return** "yes".

Proof. We start by analyzing the complexity of Algorithm 3. We assume w.l.o.g. that $|V| \geq |\mathcal{H}|$, since otherwise a factor analysis graph is trivially not a full-ZUTA graph. Computing the children of all latent nodes is of complexity at most $\mathcal{O}(|\mathcal{H}||V|)$, and ordering the latent nodes is of complexity at most $\mathcal{O}(|\mathcal{H}|^2)$. Hence, line 1 is of complexity at most $\mathcal{O}(|\mathcal{H}||V|)$. Lines 2-6 are also of complexity at most $\mathcal{O}(|\mathcal{H}||V|)$. The remaining algorithm consists of two nested for-loops, both iterating over the latent nodes. Computing the set difference in line 8 is of complexity $\mathcal{O}(|V|^2)$ and verifying membership in the set of children in line 14 is of complexity $\mathcal{O}(|V|)$. By considering the nested structure of the computations and using that $|\mathcal{H}| \leq |V|$, we conclude that the complexity of Algorithm 3 is at most $\mathcal{O}(|\mathcal{H}||V|^2)$.

Now, observe that the graph G is a full-ZUTA graph if and only if there is a relabeling

Algorithm 4 Verifying Condition (ii) of the local BB-criterion.

Input: Factor analysis graph $G = (V \cup \mathcal{H}, D)$ and a tuple $(B, S) \in 2^V \times 2^{\mathcal{H}}$ such that $G[B \cup (\text{jpa}(B) \setminus S)]$ is a full-ZUTA graph with $\prec_{\text{ZUTA}}$ being the unique ZUTA-ordering on $\text{jpa}(B) \setminus S$.

for $h \in \text{jpa}(B) \setminus S$ **do**
  Initialize $W \leftarrow \text{ch}(h) \setminus B$, $B' \leftarrow B \cap \text{ch}(h)$ and $L \leftarrow \{\ell \in \text{jpa}(B) \setminus S : \ell \preceq_{\text{ZUTA}} h\}$.
  repeat
    for $v \in W$ **do**
      for $u \in B'$ **do**
        if $\text{jpa}(\{u, v\}) \setminus S \subseteq L$ **then**
          $W \leftarrow W \setminus \{v\}$ and $B' = B' \cup \{v\}$.
          break the two inner for-loops.
        end if
      end for
    end for
  until $W = \emptyset$ or no change has occurred in the last iteration.
  if no change has occurred in the last iteration and $W \neq \emptyset$ **then**
    return “no”.
  end if
end for
return “yes”.

of the latent nodes $\mathcal{H} = \{h_1, \ldots, h_m\}$ such that (i) $|\text{ch}(h_i)| = p - i + 1$, and (ii), for all latent nodes $i = 1, \ldots, m-1$, there is an observed node $v_i \in \text{ch}(h_i)$ such that $\text{ch}(h_i) = \text{ch}(h_{i+1}) \cup \{v_i\}$. Condition (ii) holds if and only if, for all $i = 1, \ldots, m-1$, the set $W_i = \text{ch}(h_i) \setminus \text{ch}(h_{i+1})$ has cardinality $|W_i| = 1$ and the single element of W_i is not contained in $\text{ch}(h_j)$ for $j = \{i+2, \ldots, m\}$. We conclude that the output of Algorithm 3 is “yes” if and only if G is a full-ZUTA graph. □

Using Algorithm 3 we can check Condition (i) of the local BB-criterion. The purpose of the next algorithm is to check Condition (ii).

Lemma B.7. *Let $G = (V \cup \mathcal{H}, D)$ be a factor analysis graph and consider a tuple $(B, S) \in 2^V \times 2^{\mathcal{H}}$ such that $G[B \cup (\text{jpa}(B) \setminus S)]$ is a full-ZUTA graph. Then, the tuple (B, S) satisfies Condition (ii) of the local BB-criterion if and only if Algorithm 4 returns “yes”. Moreover, the algorithm has complexity at most $\mathcal{O}(|\mathcal{H}|^3 |V|^3)$.*

Algorithm 5 Deciding $\mathtt{L}(G, S)$.

Input: Factor analysis graph $G = (V \cup \mathcal{H}, D)$, and a set $S \subseteq \mathcal{H}$.

```
1: for h ∈ H \ S do
2:    for B ⊆ ch(h) such that |B| ≥ 4 do
3:       if (B, S) satisfies the local BB-criterion (Algorithms 3 and 4) then
4:          Output: "yes".
5:          break both for-loops.
6:       end if
7:    end for
8: end for
9: Output: "no".
```

Proof. It follows directly from Definition 4.14 that Algorithm 4 returns "yes" if and only if the tuple (B, S) satisfies Condition (ii) of the local BB-criterion. Hence, we only need to show the complexity. For each latent node $h \in \mathrm{jpa}(B) \setminus S$, the initialization in line 2 is of less complexity then the remaining part of the algorithm in lines 3 to 15. Another repetition in line 3 occurs only if a node was removed from W in the previous repetition. Hence, after $|W| \leq |V|$ repetitions of line 3 either all nodes were removed from W or the repetitions were stopped before. By counting the maximal number of repetitions in the for-loops and noting that checking whether $\mathrm{jpa}(\{u, v\}) \setminus S$ is a subset of L is of complexity at most $\mathcal{O}(|\mathcal{H}|^2)$, we conclude that Algorithm 4 has complexity at most $\mathcal{O}(|\mathcal{H}|^3|V|^3)$. □

To solve $\mathtt{L}(G, S)$ we need to iterate over subsets $B \subseteq V$ and, for each subset, we use Algorithms 3 and 4 to check the local BB-criterion. To shrink the number of possible subsets, we observe that there has to be a latent node $h \in \mathcal{H} \setminus S$ such that B is a subset of $\mathrm{ch}(h)$ since otherwise Condition (i) of the local BB-criterion can never be true. Hence, it is enough to first iterate over all latent nodes $h \in \mathcal{H} \setminus S$ and then iterate over all subsets $B \subseteq \mathrm{ch}(h)$ for solving $\mathtt{L}(G, S)$. The procedure for deciding $\mathtt{L}(G, S)$ is given in Algorithm 5.

Theorem B.8. *Algorithm 5 is sound and complete for deciding $\mathtt{L}(G, S)$. If we only allow sets B with $|B| \leq \ell$ in line 2, then the algorithm has complexity at most $\mathcal{O}(|\mathcal{H}|^4|V|^{\ell+3})$.*

Proof. First, we analyze the complexity of Algorithm 5. We run the "inner" algorithm (lines 2-7) at most $|\mathcal{H}|$ times. In the inner algorithm itself we iterate through subsets $B \subseteq \mathrm{ch}(h) \subseteq V$ with cardinality at most ℓ. As we have seen in the proof of Theorem B.4, the number of subsets of V with cardinality at most ℓ is $\mathcal{O}(|V|^{\ell})$. Verifying whether a given tuple (B, S) satisfies the local BB-criterion is of complexity at most $|\mathcal{H}|^3|V|^3$ as we show in Lemmas B.6 and B.7. Note that computing the joint parents of a set B is of less complexity. Hence, we conclude that the total complexity of Algorithm 5 is at most $\mathcal{O}(|\mathcal{H}|^4|V|^{\ell+3})$.

For showing that Algorithm 5 is sound and complete, it only remains to show that $B \subseteq \mathrm{ch}(h)$ for some latent node $h \in \mathcal{H}$ and $|B| \geq 4$ whenever a tuple $(B, S) \in 2^V \times 2^{\mathcal{H}}$ satisfies the local BB-criterion. Hence, suppose that $(B, S) \in 2^V \times 2^{\mathcal{H}}$ satisfies the local BB-criterion. It has to hold that $\mathrm{jpa}(B) \setminus S$ is nonempty, since otherwise Conditions (i) and (iii) of the local BB-criterion can not hold simultaneously. Moreover, if Condition (i) of the local BB-criterion holds and $G[B \cup (\mathrm{jpa}(B) \setminus S)]$ is a full-ZUTA graph, then it is easy to see that there has to be a latent node $h \in \mathcal{H}$ such that $B \subseteq \mathrm{ch}(h)$. Finally, Condition (iii) of the local BB-criterion holds if and only if $p \geq \lfloor m + \frac{1}{2}\sqrt{8m+1} + \frac{1}{2} \rfloor + 1$ for $p = |U|$ and $m = |\mathrm{jpa}(B) \setminus S|$. Since $m \geq 1$, it follows that $|U| \geq 4$. □

We conclude this section by providing the final procedure for deciding extended M-identifiability in Algorithm 6. It is easy to see that the algorithm is sound and complete. Under the same constraints as in Theorem B.4 and in Theorem B.8, the complexity is at most $\mathcal{O}(|\mathcal{H}|^2|V|^{\max\{k,l\}+1}(|V| + |\mathcal{H}|)^3)$.

Algorithm 6 Deciding extended M-identifiability.

Input: Factor analysis graph $G = (V \cup \mathcal{H}, D)$.
Initialize: Solved nodes $S \leftarrow \{h \in \mathcal{H} : \mathrm{ch}(h) = \emptyset\}$.

```
repeat
  for h ∈ H \ S do
    if M(G, h, S) holds then
      S ← S ∪ {h}.
      break
    end if
  end for
  if L(G, S) holds with B ⊆ V then
    S ← S ∪ (jpa(B) \ S)
  end if
until S = H or no change has occurred in the last iteration.
Output: "yes" if S = H, "no" otherwise.
```

C Proofs

C.1 Proof for Section 2

Proof of Lemma 2.4. It is clear by Definition 2.2 that generic sign-identifiability of the whole graph implies generic sign-identifiability of all nodes $h \in \mathcal{H}$. For the other direction, we first note that any finite intersection of Lebesgue measure zero sets is still a Lebesgue measure zero set. Now, let $(\widetilde{\Omega}, \widetilde{\Lambda}) \in \mathcal{F}_G(\Omega, \Lambda)$ be a generically chosen parameter pair. Since all nodes $h \in \mathcal{H}$ are generically sign-identifiable, it follows that $\widetilde{\Lambda} = \Lambda\Psi$, where Ψ is a $|\mathcal{H}| \times |\mathcal{H}|$ diagonal matrix with entries in $\{\pm 1\}$. It remains to show that $\widetilde{\Omega}$ is equal to Ω. By the definition of τ_G and since $\tau_G(\Omega, \Lambda) = \tau_G(\widetilde{\Omega}, \widetilde{\Lambda})$, we have that $\Omega + \Lambda\Lambda^\top = \widetilde{\Omega} + \widetilde{\Lambda}\widetilde{\Lambda}^\top$. Since $\Psi\Psi^\top$ is equal to the identity matrix, it follows that

$$\widetilde{\Omega} = \Omega + \Lambda\Lambda^\top - \widetilde{\Lambda}\widetilde{\Lambda}^\top = \Omega + \Lambda\Lambda^\top - \Lambda\Psi\Psi^\top\Lambda^\top = \Omega.$$

□

C.2 Proof for Section 3

Proof of Theorem 3.4. The original statement of the theorem in Anderson and Rubin (1956) is written as a pointwise condition for full factor analysis models. We show that the original statement is equivalent to the statement presented here. Consider a matrix $\Omega \in \mathbb{R}^{|V|}_{>0}$ and a matrix $\Lambda \in \mathbb{R}^{|V|\times|\mathcal{H}|}$. Given the matrix $\Sigma = \Lambda\Lambda^\top + \Omega$, Anderson and Rubin (1956, Theorem 5.1) states that "a sufficient condition for identification of Ω and Λ up to multiplication on the right by an orthogonal matrix is that if any row of Λ is deleted, there remain two disjoint submatrices of rank $|\mathcal{H}|$". We call this pointwise sufficient condition the "row-deletion" property. Since the row-deletion property implies identification of Ω for any pair $(\Omega, \Lambda) \in \mathbb{R}^{|V|}_{>0} \times \mathbb{R}^{|V|\times|\mathcal{H}|}$, this also holds if Λ is sparse, i.e., for pairs $(\Omega, \Lambda) \in \mathbb{R}^{|V|}_{>0} \times \mathbb{R}^{D}$.

Now, let $G = (V \cup \mathcal{H}, D)$ be a factor analysis graph such that ZUTA is satisfied and assume that for any deleted row of $\Lambda = (\lambda_{vh}) \in \mathbb{R}^D$ there exist two disjoint submatrices that are generically of rank $|\mathcal{H}|$. Hence, if $(\Omega, \Lambda) \in \Theta_G$ is generically chosen, then the row-deletion property holds and $\pi_{\text{diag}}(\mathcal{F}_G(\Omega, \Lambda)) = \{\Omega\}$, where π_{diag} is defined in (3). It follows that the graph G is generically globally identifiable and we conclude by Lemma A.2 that G is generically sign-identifiable. □

Proof of Corollary 3.11. Denote $m = |\mathcal{H}|$ and $p = |V|$. Since G is AR-identifiable, $p \geq 2m + 1$ must hold, and we have that

$$p(m+1) = \frac{1}{2}p(2m+2) \leq \frac{1}{2}p(p+1) = \frac{(p+1)!}{2!(p-1)!} = \binom{p+1}{2}.$$

Now, it holds that $\binom{m}{2} \geq 1$ since $m \geq 2$. We conclude that $|V| + |D| = p(m+1) - \binom{m}{2} < \binom{p+1}{2}$ and thus G is BB-identifiable. □

C.3 Proof for Section 4

Proof of Lemma 4.3. We first introduce some notation. Consider a collection of edges $\mathbf{M} = \{h_1 \to v_1, \dots, h_k \to v_k\} \subseteq D$, where $h_i \in \mathcal{H}$ and $v_i \in V$ for all $i = 1, \dots, k$. If all the h_i are distinct and all the v_i are distinct, then we say that $\mathbf{M}$ is a *pairing* of $S = \{h_1, \dots, h_k\}$ and $A = \{v_1, \dots, v_k\}$. Now, we mimic the proof of Sullivant et al. (2010, Lemma 3.2). By the Cauchy-Binet determinant expansion formula, we have

$$\det([\Lambda\Lambda^\top]_{A,B}) = \sum_{S \subseteq \mathcal{H}} \det(\Lambda_{A,S}) \det(\Lambda_{B,S}),$$

where the sum runs over subsets $S \subseteq \mathcal{H}$ with $|S| = |A| = |B|$. Let $M(S, A)$ be the set of all pairings of S and A. By the Lindström-Gessel–Viennot lemma (Gessel & Viennot, 1985; Lindström, 1973), $\det(\Lambda_{A,S}) = \sum_{\mathbf{M} \in M(S,A)} (-1)^{\mathbf{M}} \lambda^{\mathbf{M}}$, where $(-1)^{\mathbf{M}}$ is the sign of the induced permutation of $\mathbf{M}$ and $\lambda^{\mathbf{M}} = \prod_{h \to v \in \mathbf{M}} \lambda_{vh}$ is the monomial of edge coefficients. Since each summand $\det([\Lambda^\top]_{S,A}) \det([\Lambda^\top]_{S,B})$ consists of a sum of monomials $\lambda^{\mathbf{M}_{S,A}} \lambda^{\mathbf{M}_{S,B}}$ in different combinations of variables, the sum $\det([\Lambda\Lambda^\top]_{A,B})$ vanishes if and only if $\det(\Lambda_{A,S})$ or $\det(\Lambda_{B,S})$ is zero for all $S \subseteq |\mathcal{H}|$ with $|S| = |A| = |B|$. Now, it holds that $\det(\Lambda_{A,S})$ is zero if and only if there is no pairing of S and A. We conclude the proof by observing that the existence of a set $S \subseteq \mathcal{H}$ such that there exists a pairing of S and A and a pairing of S and B is equivalent to the existence of an intersection-free matching of A and B; also compare to Sullivant et al. (2010, Proposition 3.4). □

Proof of Corollary 4.4. Let $G = (V \cup \mathcal{H}, D)$ be a factor analysis graph such that ZUTA is satisfied. The graph G is AR-identifiable if and only if for any node $v \in V$, there are two disjoint sets of nodes $U, W \subseteq V \setminus \{v\}$ with $|W| = |U| = |\mathcal{H}|$ such that the submatrices $\Lambda_{W,\mathcal{H}}$ and $\Lambda_{U,\mathcal{H}}$ are generically of rank $|\mathcal{H}|$. This is equivalent to $\det(\Lambda_{W,\mathcal{H}})$ and $\det(\Lambda_{U,\mathcal{H}})$ not being the zero polynomials which holds if and only if $\det([\Lambda\Lambda^\top]_{W,U})$ is also not the

zero polynomial. Finally, by Lemma 4.3, the determinant $\det([\Lambda\Lambda^\top]_{W,U})$ is not the zero polynomial if and only if there is an intersection-free matching between W and U. $\square$

Proof of Theorem 4.8. Fix a generically chosen parameter tuple $(\Omega, \Lambda) \in \Theta_G$ and let $\Sigma = (\sigma_{ij}) = \tau_G(\Omega, \Lambda)$ be its image in $PD(|V|)$. Consider a tuple $(\widetilde{\Omega}, \widetilde{\Lambda}) \in \mathcal{F}_G(\Omega, \Lambda)$ in the fiber of (Ω, Λ). Since all nodes $\ell \in S$ are generically sign-identifiable, it holds that $\widetilde{\Lambda}_{\text{ch}(\ell),\ell} = a_\ell \Lambda_{\text{ch}(\ell),\ell}$ for some $a_\ell \in \{\pm 1\}$. We have to show that the vector $\widetilde{\Lambda}_{\text{ch}(h),h}$ also coincides with $\Lambda_{\text{ch}(h),h}$ up to sign. Define

$$\widehat{\sigma}_{uw} = \sigma_{uw} - \sum_{\ell \in \text{jpa}(\{u,w\}) \cap S} \widetilde{\lambda}_{u,\ell} \widetilde{\lambda}_{w,\ell},$$

for all $u, w \in V$. Here, $\widetilde{\lambda}_{u,\ell}$ denotes the entry of $\widetilde{\Lambda}$ that is indexed by row u and column ℓ. Since $\widetilde{\Lambda}_{\text{ch}(\ell),\ell} = a_\ell \Lambda_{\text{ch}(\ell),\ell}$, we have for $u \neq w$ that

$$\begin{aligned} \widehat{\sigma}_{uw} &= \sum_{\ell \in \text{jpa}(\{u,w\})} \lambda_{u,\ell}\lambda_{w,\ell} - \sum_{\ell \in \text{jpa}(\{u,w\}) \cap S} a_\ell^2 \lambda_{u,\ell}\lambda_{w,\ell} \\ &= \sum_{\ell \in \text{jpa}(\{u,w\}) \setminus S} \lambda_{u,\ell}\lambda_{w,\ell}. \end{aligned} \tag{5}$$

Since $(\widetilde{\Omega}, \widetilde{\Lambda}) \in \mathcal{F}_G(\Omega, \Lambda)$, we also have $\Sigma = \tau_G(\widetilde{\Omega}, \widetilde{\Lambda})$. Hence, it also holds that

$$\begin{aligned} \widehat{\sigma}_{uw} &= \sum_{\ell \in \text{jpa}(\{u,w\})} \widetilde{\lambda}_{u,\ell}\widetilde{\lambda}_{w,\ell} - \sum_{\ell \in \text{jpa}(\{u,w\}) \cap S} \widetilde{\lambda}_{u,\ell}\widetilde{\lambda}_{w,\ell} \\ &= \sum_{\ell \in \text{jpa}(\{u,w\}) \setminus S} \widetilde{\lambda}_{u,\ell}\widetilde{\lambda}_{w,\ell}, \end{aligned} \tag{6}$$

Observe that the matrix $\widehat{\Sigma} = (\widehat{\sigma}_{uv})$ lies in the model $F(\widehat{G})$, where the augmented factor analysis graph $\widehat{G} = (V \cup \widehat{\mathcal{H}}, \widehat{D})$ is obtained from G by removing the nodes $\ell \in S$ and their adjacent edges, that is, $\widehat{\mathcal{H}} = \mathcal{H} \setminus S$ and $\widehat{D} = \{h \to v \in D : h \in \widehat{\mathcal{H}}\}$. We define the following $(k+1) \times (k+1)$ matrix:

$$A = \left(\begin{array}{c|c} \lambda_{v,h}^2 & \widehat{\Sigma}_{v,U} \\ \hline \widehat{\Sigma}_{W,v} & \widehat{\Sigma}_{W,U} \end{array}\right).$$

Since $\mathrm{pa}(v) \setminus S = \{h\}$ and due to Equation (5) it holds that $A = (\widehat{\Lambda}\widehat{\Lambda}^\top)_{\{v\}\cup W, \{v\}\cup U}$ with $\widehat{\Lambda} = (\lambda_{u\ell}) \in \mathbb{R}^{\widehat{D}}$. Now, by the definition of the matching criterion, there does not exist an intersection-free matching of $\{v\} \cup W$ and $\{v\} \cup U$ in the graph G that avoids S. Hence, every matching of $\{v\} \cup W$ and $\{v\} \cup U$ in the graph $\widehat{G}$ is also not intersection-free. By Lemma 4.3 we conclude that $\det(A) = 0$, that is, the determinant is equal to the zero polynomial. Expansion among the first row yields

$$0 = \det(A) = \lambda_{v,h}^2 \det(\widehat{\Sigma}_{W,U}) - \underbrace{\sum_{i=1}^{k} (-1)^i \widehat{\sigma}_{vu_i} \det(\widehat{\Sigma}_{W,\{v\}\cup(U\setminus\{u_i\})})}_{=:B},$$

where $U = \{u_1, \ldots, u_k\}$. In order to solve this equation for $\lambda_{v,h}^2$ it has to hold that $\det(\widehat{\Sigma}_{W,U})$ is not zero. Indeed, by the definition of the matching criterion, we have that there is an intersection-free matching of W and U. Since this matching avoids the set S in the graph G, it is also an intersection-free matching in the graph $\widehat{G}$. In particular, $\det(\widehat{\Sigma}_{W,U})$ is not the zero polynomial. Since $\Lambda \in \mathbb{R}^D$ was generically chosen, we conclude that $\det(\widehat{\Sigma}_{W,U}) \neq 0$ and we obtain

$$\lambda_{v,h}^2 = B / \det(\widehat{\Sigma}_{W,U}). \tag{7}$$

Now, define $\widetilde{A}$ equivalent as A but replace $\lambda_{v,h}^2$ in the upper left corner with $\widetilde{\lambda}_{v,h}^2$. Recalling Equation (6) and by repeating the same arguments, we also obtain

$$\widetilde{\lambda}_{v,h}^2 = B / \det(\widehat{\Sigma}_{W,U}). \tag{8}$$

Taking Equations (7) and (8) together, it follows that $\widetilde{\lambda}_{v,h} = a_h \lambda_{v,h}$ for some $a_h \in \{\pm 1\}$. For the remaining children $u \in \mathrm{ch}(h) \setminus \{v\}$, recall from Equations (5) and (6) that $\widehat{\sigma}_{vu} = \lambda_{v,h}\lambda_{u,h} = \widetilde{\lambda}_{v,h}\widetilde{\lambda}_{u,h}$. Dividing by $\widetilde{\lambda}_{v,h}$ yields

$$\widetilde{\lambda}_{u,h} = \frac{\lambda_{v,h}\lambda_{u,h}}{\widetilde{\lambda}_{v,h}} = a_h \frac{\lambda_{v,h}\lambda_{u,h}}{\lambda_{v,h}} = a_h \lambda_{u,h},$$

which is also well-defined for generic parameter choices. We conclude that $\widetilde{\Lambda}_{\mathrm{ch}(h),h} = a_h\Lambda_{\mathrm{ch}(h),h}$, as claimed. Finally, note that that the set of points $(\Omega, \Lambda) \in \mathbb{R}^{|V|+|D|}$, where $\det(\widehat{\Sigma}_{W,U})$ or $\lambda_{v,h}$ is zero, defines a proper algebraic subset. Hence, it is a null set in Θ_G; see e.g. the lemma in Okamoto (1973). □

Proof of Corollary 4.10. We begin by showing statement (i). Let $G = (V \cup \mathcal{H}, D)$ be a factor analysis graph that satisfies ZUTA and is AR-identifiable. Let $\prec$ be a ZUTA-ordering on $\mathcal{H}$ with respect to G. Fix a latent node $h \in \mathcal{H}$ and assume that all nodes $\ell \in \mathcal{H}$ with $\ell \prec h$ are generically sign-identifiable. To show that G is M-identifiable, it is enough to show that there are $v \in V$ and $W, U \subseteq V$ such that the tuple (v, W, U, S) with $S = \{\ell \in \mathcal{H} : \ell \prec h\}$ satisfies the matching criterion with respect to h.

Since G satisfies ZUTA, there is an observed node $v \in \mathrm{ch}(h)$ such that $v \in \mathrm{ch}(h)$ and $v \notin \bigcup_{\ell \succ h} \mathrm{ch}(\ell)$. Hence, $\mathrm{pa}(v) \setminus S = \{h\}$. By Corollary 4.4, there further exist two disjoint sets $\widetilde{W}, \widetilde{U} \subseteq V \setminus \{v\}$ with $|\widetilde{W}| = |\widetilde{U}| = |\mathcal{H}|$ such that there is an intersection-free matching between $\widetilde{W}$ and $\widetilde{U}$. Define $\widetilde{W} = \{w_1, \ldots, w_{|\mathcal{H}|}\}$ and $\widetilde{U} = \{u_1, \ldots, u_{|\mathcal{H}|}\}$ and let $\Pi = \{\pi_1, \ldots, \pi_{|\mathcal{H}|}\}$ be an intersection-free matching of $\widetilde{W}$ and $\widetilde{U}$ such that π_i is given by $w_i \leftarrow h_i \rightarrow u_i$. Note that the set of latent nodes $\{h_1, \ldots, h_{|\mathcal{H}|}\}$ that appear in the matching Π is equal to the set of all latent nodes $\mathcal{H}$ since the matching is intersection-free. If a latent node $h_i \in S$, we remove w_i from $\widetilde{W}$ and u_i from $\widetilde{U}$. This defines W and U as subsets of $\widetilde{W}$ and $\widetilde{U}$ respectively. Note that the sets W and U are nonempty since $h \notin S$.

To see that the tuple (v, W, U, S) indeed satisfies the matching criterion, the only non-trivial condition that remains to be checked is condition (iv) of Definition 4.7. Suppose there is an intersection free matching of $\{v\} \cup W$ and $\{v\} \cup U$ that avoids S. Adding the paths $\pi_i : w_i \leftarrow h_i \rightarrow u_i$ with $h_i \in S$ to this matching, gives an intersection-free matching of $\{v\} \cup \widetilde{W}$ and $\{v\} \cup \widetilde{U}$. On the other hand, any matching of $\{v\} \cup \widetilde{W}$ and $\{v\} \cup \widetilde{U}$ must

intersect since $|\widetilde{W}| = |\widetilde{U}| = |\mathcal{H}|$. We conclude by contradiction that there can not exist an intersection-free matching of $\{v\} \cup W$ and $\{v\} \cup U$ that avoids S.

We now show statement (ii). One direction follows from (i). For the other direction, let $G = (V \cup \mathcal{H}, D)$ be a full-ZUTA graph that is M-identifiable. By Corollary 4.4, we need to show that, for any $v \in V$, there exist two disjoint sets $W, U \subseteq V \setminus \{v\}$ with $|W| = |U| = |\mathcal{H}|$ such that there is an intersection-free matching between W and U. If the latter condition is satisfied for a node $v \in V$, we say for simplicity that U and W *satisfy the AR-condition* for the node v.

Since G is M-identifiable, there is a relabeling of the latent nodes $\mathcal{H} = \{h_1, \ldots, h_m\}$ and there are tuples $(v_j, W_j, U_j, S_j) \in V \times 2^V \times 2^V \times 2^{\mathcal{H} \setminus \{h_j\}}$ that satisfy the matching criterion with respect to h_j, such that $i < j$ whenever $h_i \in S_j$. For the first latent node h_1, it must hold that $S_1 = \emptyset$. Moreover, since G is a full-ZUTA graph, we can relabel the elements of U_1 and relabel the elements of W_1 such that $U_1 = \{u_1, \ldots, u_s\}$ and $W_1 = \{w_1, \ldots, w_s\}$, and $h_{k+1} \in \mathrm{jpa}(w_k, u_k)$ for $k = 1, \ldots, s$ and $s \in [|\mathcal{H}|]$.

First, we observe that $|U_1| = |W_1| = s = |\mathcal{H}|$. Assume that this is not true, i.e., $|U_1| = |W_1| = s < |\mathcal{H}|$. Then there is an intersection-free matching of $\{v_1\} \cup U_1$ and $\{v_1\} \cup W_1$ given by the paths $v_1 \leftarrow h_1 \leftarrow v_1$ and $u_k \leftarrow h_{k+1} \leftarrow v_k$ for all $k = 1, \ldots, s$, which is a contradiction to property (iv) of the matching criterion. We conclude that $|U_1| = |W_1| = |\mathcal{H}|$ and, in particular, the AR-condition is satisfied by $U = U_1$ and $W = W_1$ for the node v_1. By taking $U = U_1$ and $W = W_1$, the AR-condition is also satisfied for all $v \in V \setminus (U_1 \cup W_1 \cup \{v_1\})$.

It remains to show that there exist U and W such that the AR-condition is satisfied for $v \in U_1 \cup W_1$. W.l.o.g. we may assume that $v = u_k \in U_1$. Take $U = (U_1 \setminus u_k) \cup \{v_1\}$ and $W = W_1$. Since $h_{k+1} \in \mathrm{jpa}(w_k, u_k)$, there exists an intersection-free matching of U and W

given by the paths $v_1 \leftarrow h_1 \rightarrow w_1$, $u_{i-1} \leftarrow h_i \rightarrow w_i$ for all $i = 2, \ldots, k$ and $u_i \leftarrow h_i \rightarrow w_i$ for all $i = k+1, \ldots, |\mathcal{H}|$. $\square$

Proof of Corollary 4.11. Suppose that $G = (V \cup \mathcal{H}, D)$ is M-identifiable. Then there is a total ordering $\prec$ on the latent nodes $\mathcal{H}$ such that $\ell \prec h$ whenever $\ell \in S_h$, where $(v_h, W_h, U_h, S_h) \in V \times 2^V \times 2^V \times 2^{\mathcal{H}\setminus\{h\}}$ satisfies the matching criterion with respect to h. It follows that $\ell \prec h$ if $\ell \in \mathrm{pa}(v_h) \setminus \{h\}$. Said differently, $v_h \in \mathrm{ch}(h)$ but $v_h \notin \mathrm{ch}(\ell)$ if $h \prec \ell$. We conclude that the ordering $\prec$ is a ZUTA-ordering with respect to G. $\square$

Proof of Theorem 4.15. Fix a generically chosen parameter tuple $(\Omega, \Lambda) \in \Theta_G$ and let $\Sigma = (\sigma_{ij}) = \tau_G(\Omega, \Lambda)$ be its image in $PD(|V|)$. Consider a tuple $(\widetilde{\Omega}, \widetilde{\Lambda}) \in \mathcal{F}_G(\Omega, \Lambda)$ in the fiber of (Ω, Λ). By assumption, for all nodes $\ell \in S$, it holds that $\widetilde{\Lambda}_{\mathrm{ch}(\ell),\ell} = a_\ell \Lambda_{\mathrm{ch}(\ell),\ell}$ for some $a_\ell \in \{\pm 1\}$. We have to show for all $h \in \mathrm{jpa}(B) \setminus S$ that $\widetilde{\Lambda}_{\mathrm{ch}(h),h}$ also coincides with $\Lambda_{\mathrm{ch}(h),h}$ up to sign. As in the proof of Theorem 4.8, define

$$\widehat{\sigma}_{vw} = \sigma_{vw} - \sum_{\ell \in \mathrm{jpa}(\{v,w\}) \cap S} \widetilde{\lambda}_{v,\ell} \widetilde{\lambda}_{w,\ell},$$

for all $v, w \in V$. Since $\widetilde{\Lambda}_{\mathrm{ch}(\ell),\ell} = a_\ell \Lambda_{\mathrm{ch}(\ell),\ell}$, we have for $v \neq w$ that

$$\begin{aligned}\widehat{\sigma}_{vw} &= \sum_{\ell \in \mathrm{jpa}(\{v,w\})} \lambda_{v,\ell}\lambda_{w,\ell} - \sum_{\ell \in \mathrm{jpa}(\{v,w\}) \cap S} a_\ell^2 \lambda_{v,\ell}\lambda_{w,\ell} \\ &= \sum_{\ell \in \mathrm{jpa}(\{v,w\}) \setminus S} \lambda_{v,\ell}\lambda_{w,\ell}.\end{aligned}$$

Since $(\widetilde{\Omega}, \widetilde{\Lambda}) \in \mathcal{F}_G(\Omega, \Lambda)$, we also have $\Sigma = \tau_G(\widetilde{\Omega}, \widetilde{\Lambda})$. Hence, it also holds that

$$\begin{aligned}\widehat{\sigma}_{vw} &= \sum_{\ell \in \mathrm{jpa}(\{v,w\})} \widetilde{\lambda}_{v,\ell}\widetilde{\lambda}_{w,\ell} - \sum_{\ell \in \mathrm{jpa}(\{v,w\}) \cap S} \widetilde{\lambda}_{v,\ell}\widetilde{\lambda}_{w,\ell} \\ &= \sum_{\ell \in \mathrm{jpa}(\{v,w\}) \setminus S} \widetilde{\lambda}_{v,\ell}\widetilde{\lambda}_{w,\ell}.\end{aligned}$$

Observe that the submatrix $\widehat{\Sigma}_{B,B}$ of the matrix $\widehat{\Sigma} = (\widehat{\sigma}_{vw})$ lies in the model $F(\widetilde{G})$, where $\widetilde{G} = G[B \cup (\mathrm{jpa}(B) \setminus S)]$. Let $\mathcal{L} = \mathrm{jpa}(B) \setminus S$ be the latent nodes of $\widetilde{G}$, and let $\widetilde{D}$ be

the edge set of $\widetilde{G}$. Since $\widetilde{G}$ is a full-ZUTA graph and $|B| + |\widetilde{D}| < \binom{|B|+1}{2}$, it follows from Theorem 3.7 that $\widetilde{G}$ is generically sign-identifiable. Recalling Definition 2.2, this means that

$$\widetilde{\Lambda}_{B,\mathcal{L}} = \Lambda_{B,\mathcal{L}}\Psi, \tag{9}$$

where $\Psi \in \mathbb{R}^{|\mathcal{L}|\times|\mathcal{L}|}$ is a diagonal matrix with diagonal entries in $\{\pm 1\}$. It remains to show that Equation (9) also holds for all nodes in $V \setminus B$, i.e., we need to show that $\widetilde{\Lambda}_{V,\mathcal{L}} = \Lambda_{V,\mathcal{L}}\Psi$. We will show this by a double induction: We first induct on the unique ZUTA-ordering $\prec_{\text{ZUTA}}$ of the latent nodes $\mathcal{L}$ with respect to $\widetilde{G}$. Within each induction step, where we consider a fixed node $h \in \mathcal{L}$, we then induct on the U-first-ordering $\prec_h$ on $\text{ch}(h)$.

Let $h \in \mathcal{L}$ and assume that $\widetilde{\Lambda}_{V,\ell} = a_\ell \Lambda_{V,\ell}$ for all $\ell \prec_{\text{ZUTA}} h$, where $a_\ell \in \{\pm 1\}$. Moreover, consider a node $v \in \text{ch}(h)$. For the base case of the induction, we note that $B \cap \text{ch}(h) \neq \emptyset$ because of Conditions (i) and (iii) of the local BB-criterion. By Equation (9), it holds for all $v \in \text{ch}(h) \cap B$ that $\widetilde{\lambda}_{v,h} = a_h \lambda_{v,h}$, where $a_h = \Psi_{hh} \in \{\pm 1\}$.

Now, let $v \in \text{ch}(h) \setminus B$ and assume that $\widetilde{\lambda}_{u,h} = a_h \lambda_{u,h}$ whenever $u \prec_h v$ for $u \in \text{ch}(h)$. By property (ii) of the local BB-criterion, there has to be $u \in \text{ch}(h)$ with $u \prec_h v$ such that $\text{jpa}(\{v, u\}) \setminus S \subseteq \{\ell \in \mathcal{L} : \ell \preceq_{\text{ZUTA}} h\}$. Define $T = S \cup \{\ell \in \mathcal{L} : \ell \prec_{\text{ZUTA}} h\}$ and consider the quantity

$$\overline{\sigma}_{vu} = \sigma_{vu} - \sum_{\ell \in \text{jpa}(v,u) \cap T} \widetilde{\lambda}_{v,\ell} \widetilde{\lambda}_{u,\ell}.$$

Observe $\text{jpa}(v, u) \setminus T = \{h\}$. Since $\Sigma = \tau_G(\Omega, \Lambda) = \tau_G(\widetilde{\Omega}, \widetilde{\Lambda})$, it follows similarly as above that

$$\overline{\sigma}_{vu} = \lambda_{v,h}\lambda_{u,h} = \widetilde{\lambda}_{v,h}\widetilde{\lambda}_{u,h}.$$

Dividing by $\widetilde{\lambda}_{u,h} = a_h \lambda_{u,h}$ yields

$$\widetilde{\lambda}_{v,h} = \frac{\lambda_{v,h}\lambda_{u,h}}{\widetilde{\lambda}_{u,h}} = a_h \frac{\lambda_{v,h}\lambda_{u,h}}{\lambda_{u,h}} = a_h \lambda_{v,h},$$

which is well-defined for generic parameter choices. We conclude that $\widetilde{\Lambda}_{\text{ch}(h),h} = a_h \Lambda_{\text{ch}(h),h}$.

□

Proof of Corollary 4.17. Let $G = (V \cup \mathcal{H}, D)$ be a full-ZUTA graph that is BB-identifiable. It is enough to show that the tuple $(B, S) = (V, \emptyset)$ satisfies the local BB-criterion. Condition (i) is satisfied since BB-identifiability implies $|\mathcal{H}| < |V|$, and hence $\text{jpa}(V) = \mathcal{H}$, which yields that the induced subgraph $\widetilde{G} = G[V \cup \text{jpa}(V)]$ is equal to G. It follows that Condition (iii) is also satisfied since BB-identifiability implies $|V| + |D| < \binom{p+1}{2}$. To conclude, observe that there is nothing to show in Condition (ii) since $\text{ch}(h) \setminus V = \emptyset$ for all $h \in \mathcal{H}$.

For the other direction, let $G = (V \cup \mathcal{H}, D)$ be a full-ZUTA graph and suppose that we can certify generic sign-identifiability of G by recursively applying Theorem 4.15. It is enough to show that whenever the local BB-criterion is satisfied in a full-ZUTA graph for some tuple $(B, S) \in 2^V \times 2^{\mathcal{H}}$ with $S = \emptyset$, then it must hold that $\text{jpa}(B) = \mathcal{H}$. Since the induced graph $G[B \cup \mathcal{H}]$ is a full-ZUTA graph and Condition (iii) holds, we can then replace B by V and Condition (iii) still holds, i.e., the graph G is BB-identifiable.

Let $\widetilde{D}$ be the edge set of the induced subgraph $G[B \cup \text{jpa}(B)]$ To show that $\text{jpa}(B) = \mathcal{H}$, we first observe that $\text{jpa}(B)$ can not be the empty set since in this case $|B| \leq 1$ and hence Condition (iii) does not hold. Thus it must be that $|B| > 1$ and $|\text{jpa}(B)| \geq 1$. It holds that $|B| + |\widetilde{D}| < \binom{|B|+1}{2}$ if and only if $|B| \geq \lfloor |\text{jpa}(B)| + \frac{1}{2}\sqrt{8|\text{jpa}(B)| + 1} + \frac{1}{2} \rfloor + 1$. Since $|\text{jpa}(B)| \geq 1$, this implies, in particular, that $|B| \geq |\text{jpa}(B)| + 2$. Now, suppose $\text{jpa}(B) \neq \mathcal{H}$. Since $|B| \geq |\text{jpa}(B)| + 2$ and G is a full-ZUTA graph, there must be two nodes $u, w \in B$ such that there is a latent node $h \in \text{jpa}(\{u, w\}) \setminus \text{jpa}(B)$. This is a contradiction and we conclude that $\text{jpa}(B) = \mathcal{H}$.

□

D Deciding Identifiability by Computational Algebra

Generic sign-identifiability may be decided by computational algebraic geometry. We make use of the following lemma where, for a symmetric matrix $M \in \mathbb{R}^{p \times p}$, we denote by $\mathrm{od}(M) \in \mathbb{R}^{\binom{p}{2}}$ the vector of off-diagonal entries of M.

Lemma D.1. *A factor analysis graph $G = (V \cup \mathcal{H}, D)$ is generically sign-identifiable if and only if the map*

$$\phi_G : \mathbb{R}^D \longrightarrow \mathbb{R}^{\binom{|V|}{2}}$$

$$\Lambda \longmapsto od(\Lambda\Lambda^\top)$$

has fibers of the form

$$\phi_G^{-1}(\phi_G(\Lambda)) = \{\widetilde{\Lambda} \in \mathbb{R}^D : \widetilde{\Lambda} = \Lambda\Psi \text{ for } \Psi \in \{\pm 1\}^{|\mathcal{H}| \times |\mathcal{H}|} \text{ diagonal}\}$$

for almost all $\Lambda \in \mathbb{R}^D$.

Proof. Let $G = (V \cup \mathcal{H}, D)$ be a factor analysis graph. Take a generic tuple $(\Omega, \Lambda) \in \Theta_G$, and assume that the fiber $\phi_G^{-1}(\phi_G(\Lambda))$ is of the form as in the statement. Now, consider another tuple $(\widetilde{\Omega}, \widetilde{\Lambda}) \in \mathcal{F}_G(\Omega, \Lambda)$. Since $\Omega + \Lambda\Lambda^\top = \widetilde{\Omega} + \widetilde{\Lambda}\widetilde{\Lambda}^\top$ and Ω and $\widetilde{\Omega}$ are diagonal, we also have the equality $\mathrm{od}(\Lambda\Lambda^\top) = \mathrm{od}(\widetilde{\Lambda}\widetilde{\Lambda}^\top)$. It follows that $\widetilde{\Lambda} = \Lambda\Psi$, where Ψ is a $|\mathcal{H}| \times |\mathcal{H}|$ diagonal matrix with entries in $\{\pm 1\}$. But then we also have that

$$\widetilde{\Omega} = \Omega + \Lambda\Lambda^\top - \widetilde{\Lambda}\widetilde{\Lambda}^\top = \Omega + \Lambda\Lambda^\top - \Lambda\Psi\Psi^\top\Lambda^\top = \Omega,$$

where we have used that $\Psi\Psi^\top$ is equal to the identity matrix. Hence, we have shown that G is generically sign-identifiable.

For the other direction, consider a generic matrix $\Lambda \in \mathbb{R}^D$ and assume that G is generically sign-identifiable. For any matrix $\widetilde{\Lambda} \in \phi_G^{-1}(\phi_G(\Lambda))$, it holds that $\mathrm{od}(\Lambda\Lambda^\top) = \mathrm{od}(\widetilde{\Lambda}\widetilde{\Lambda}^\top)$.

Hence, $\Lambda\Lambda^\top - \widetilde{\Lambda}\widetilde{\Lambda}^\top = D$, where D is a diagonal matrix. Now, consider another diagonal matrix Q that is positive definite with $\min_{v\in V} Q_{vv} > -\min_{v\in V} D_{vv}$ if $\min_{v\in V} D_{vv}$ is negative. We have the equality

$$\Lambda\Lambda^\top + Q = \widetilde{\Lambda}\widetilde{\Lambda}^\top + D + Q,$$

where, by construction, both Q and $D+Q$ are positive definite. Note that Q can be chosen from a set that has positive measure in $\mathbb{R}^{|V|}$. By the generic sign-identifiability of G we can thus conclude that $D = 0$ and that $\widetilde{\Lambda} = \Lambda\Psi$, where Ψ is a $|\mathcal{H}| \times |\mathcal{H}|$ diagonal matrix with entries in $\{\pm 1\}$. □

Let $\mathcal{F}_\phi(\Lambda) = \{\widetilde{\Lambda} \in \mathbb{R}^D : \phi_G(\widetilde{\Lambda}) = \phi_G(\Lambda)\}$ be the fiber of a matrix $\Lambda \in \mathbb{R}^D$ under the map ϕ_G given in Lemma D.1. Moreover, we denote by $\mathcal{F}_{\phi,\mathbb{C}}(\Lambda) = \{\widetilde{\Lambda} \in \mathbb{C}^D : \phi_G(\widetilde{\Lambda}) = \phi_G(\Lambda)\}$ the complex fiber under the extension of the map ϕ_G to the domain $\mathbb{C}^D$. In the following, we explain how to obtain the complex fiber $\mathcal{F}_{\phi,\mathbb{C}}(\Lambda_0)$ of a generically chosen parameter point $\Lambda_0 \in \mathbb{R}^D$ by computational algebra, and we discuss how this may allow us to determine generic sign-identifiability over the real numbers.

We denote by $\mathbb{R}[\lambda_{vh} : h \to v \in D]$ the ring over the indeterminates corresponding to the edges in the graph G with real coefficients. Moreover, from now on, we let $\Lambda \in \mathbb{R}^D$ be the matrix with entries Λ_{vh} being the indeterminates λ_{vh} whenever $h \to v \in D$ and zero otherwise. For a randomly chosen matrix $\Lambda_0 \in \mathbb{R}^D$, we compute a reduced Gröbner basis for the equation system

$$\phi_G(\Lambda_0) - \mathrm{od}(\Lambda\Lambda^\top) \tag{10}$$

with an arbitrary term order on the indeterminates λ_{vh}; see Cox, Little, and O'Shea (2007) for background on Gröbner bases. The reduced Gröbner basis allows us to compute both

fibers $\mathcal{F}_{\phi,\mathbb{C}}(\Lambda_0)$ and $\mathcal{F}_{\phi}(\Lambda_0)$. If Λ_0 is drawn from a continuous probability distribution, then the dimension and cardinality of the complex fibers $\mathcal{F}_{\phi,\mathbb{C}}(\Lambda_0)$ coincide with probability one. However, this is not true for the real fibers $\mathcal{F}_{\phi}(\Lambda_0)$.

We now explain how we determine generic-sign identifiability when knowing the complex fiber $\mathcal{F}_{\phi,\mathbb{C}}(\Lambda_0)$ of a generically chosen parameter matrix $\Lambda_0 \in \mathbb{R}^D$. Let $\mathbf{\Pi} := \{\pm 1\}^{|\mathcal{H}|\times|\mathcal{H}|}$ be the group of diagonal matrices with diagonal entries in $\{\pm 1\}$. For $\Lambda, \widetilde{\Lambda} \in \mathbb{C}^D$, we define the equivalence relation

$$\Lambda \sim \widetilde{\Lambda} \text{ if there is } \Psi \in \mathbf{\Pi} \text{ such that } \widetilde{\Lambda}\Psi = \Lambda,$$

and denote by $\mathcal{F}_{\phi,\mathbb{C}}(\Lambda_0)/\mathbf{\Pi}$ the set of equivalence classes of $\mathcal{F}_{\phi,\mathbb{C}}(\Lambda_0)$. Note that for a generically chosen point $\Lambda_0 \in \mathbb{R}^D$, the number of complex equivalence classes $|\mathcal{F}_{\phi,\mathbb{C}}(\Lambda_0)/\mathbf{\Pi}|$ is always the same.

Definition D.2. Let $G = (V \cup \mathcal{H}, D)$ be a factor analysis graph and let $\Lambda_0 \in \mathbb{R}^D$ be a generic parameter matrix. We say that the number of complex equivalence classes $|\mathcal{F}_{\phi,\mathbb{C}}(\Lambda_0)/\mathbf{\Pi}| \in \mathbb{N} \cup \{\infty\}$ is the *degree of sign-identifiability*.

To guard against false conclusions, we repeat the randomized calculations to determine the degree of sign-identifiability several times for each graph in practice. For computing a reduced Gröbner basis one can use any computer algebra system such as SINGULAR (Decker, Greuel, Pfister, & Schönemann, 2024), Macaulay2 (Grayson & Stillman, 2024) or SageMath (Stein, 2024). A factor analysis graph is generically sign-identifiable if its degree of sign-identifiability is 1. If the degree of sign-identifiability is infinite, then the real fiber $\mathcal{F}_{\phi}(\Lambda)$ is also infinite (Whitney, 1957, Lemma 9). If the degree of identifiability is a finite number larger or equal to 2, then generic sign-identifiability may or may not hold. Formally, one needs to verify that the set of $\Lambda_0 \in \mathbb{R}^D$ where the number of real equivalence classes

$|\mathcal{F}_\phi(\Lambda_0)/\mathbf{\Pi}|$ is larger or equal than 2 has a nonzero measure. In practice, we compute the number $|\mathcal{F}_\phi(\Lambda_0)/\mathbf{\Pi}|$ for several random draws of $\Lambda_0 \in \mathbb{R}^D$ and conclude that the graph is not generically sign-identifiable if we find $|\mathcal{F}_\phi(\Lambda_0)/\mathbf{\Pi}| \geq 2$ for at least two random draws of Λ_0. In our experiments in Section 6 we always found that if the degree of identifiability is a finite number larger or equal to 2, then the graph is not generically sign-identifiable.

References

Anderson, T. W., & Rubin, H. (1956). Statistical inference in factor analysis. In *Proceedings of the Third Berkeley Symposium on Mathematical Statistics and Probability, 1954–1955, vol. V* (pp. 111–150). Univ. California Press.

Barber, R. F., Drton, M., Sturma, N., & Weihs, L. (2022). Half-trek criterion for identifiability of latent variable models. *The Annals of Statistics*, *50*(6), 3174–3196. doi: 10.1214/22-aos2221

Bekker, P. A., & ten Berge, J. M. F. (1997). Generic global indentification in factor analysis. *Linear Algebra and its Applications*, *264*, 255–263. doi: 10.1016/S0024-3795(96)00363-1

Cormen, T. H., Leiserson, C. E., Rivest, R. L., & Stein, C. (2009). *Introduction to algorithms* (Third ed.). MIT Press.

Cox, D., Little, J., & O'Shea, D. (2007). *Ideals, varieties, and algorithms* (Third ed.). Springer, New York. doi: 10.1007/978-0-387-35651-8

Decker, W., Greuel, G.-M., Pfister, G., & Schönemann, H. (2024). Singular*, a computer algebra system for polynomial computations (Version 4.4.0).* Retrieved from http://www.singular.uni-kl.de

Ford, L. R., Jr., & Fulkerson, D. R. (1962). *Flows in networks.* Princeton University Press.

Foygel, R., Draisma, J., & Drton, M. (2012). Half-trek criterion for generic identifiability of linear structural equation models. *The Annals of Statistics*, *40*(3), 1682–1713. doi: 10.1214/12-AOS1012

Gessel, I., & Viennot, G. (1985). Binomial determinants, paths, and hook length formulae. *Advances in Mathematics*, *58*(3), 300–321. doi: 10.1016/0001-8708(85)90121-5

Grayson, D. R., & Stillman, M. E. (2024). *Macaulay2, a software system for research in algebraic geometry (Version 1.24.11).* Retrieved from http://www2.macaulay2.com

Lindström, B. (1973). On the vector representations of induced matroids. *The Bulletin of the London Mathematical Society*, *5*, 85–90. doi: 10.1112/blms/5.1.85

Okamoto, M. (1973). Distinctness of the eigenvalues of a quadratic form in a multivariate sample. *The Annals of Statistics*, *1*, 763–765. doi: 10.1214/aos/1176342

Stein, W. A. e. a. (2024). *Sage Mathematics Software (Version 10.4).* Retrieved from https://www.sagemath.org/

Sullivant, S., Talaska, K., & Draisma, J. (2010). Trek separation for Gaussian graphical models. *The Annals of Statistics*, *38*(3), 1665–1685. doi: 10.1214/09-AOS760

Whitney, H. (1957). Elementary structure of real algebraic varieties. *Annals of Mathematics. Second Series*, *66*, 545–556. doi: 10.2307/1969908
